# High-Performance Data Processing Workflow Incorporating Effect-Directed Analysis for Feature Prioritization in Suspect and Nontarget Screening

**DOI:** 10.1021/acs.est.1c04168

**Published:** 2022-01-20

**Authors:** Tim J. H. Jonkers, Jeroen Meijer, Jelle J. Vlaanderen, Roel C. H. Vermeulen, Corine J. Houtman, Timo Hamers, Marja H. Lamoree

**Affiliations:** † Department of Environment & Health, Faculty of Science, Amsterdam Institute of Molecular and Life Sciences, Vrije Universiteit Amsterdam, De Boelelaan 1085, 1081 HV Amsterdam, The Netherlands; ‡ Institute for Risk Assessment Sciences (IRAS), Utrecht University, Yalelaan 2, 3584 CM Utrecht, the Netherlands; § The Water Laboratory, J.W. Lucasweg 2, 2031 BE Haarlem, The Netherlands

**Keywords:** effect-directed analysis, bioassay, suspect and nontarget screening, environment, antibiotic, TTR-binding

## Abstract

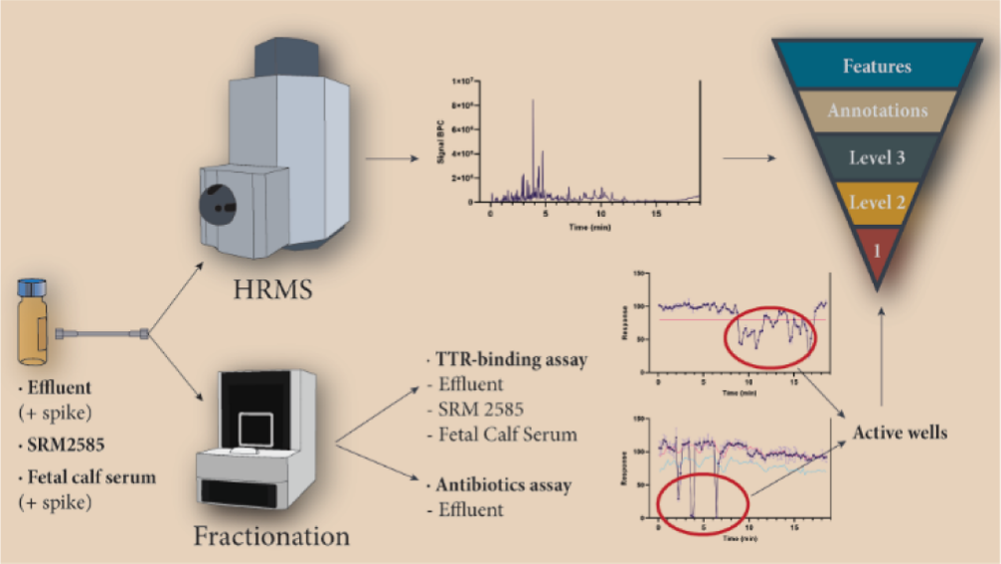

Effect-directed analysis (EDA) aims at the detection of bioactive chemicals of emerging concern (CECs) by combining toxicity testing and high-resolution mass spectrometry (HRMS). However, consolidation of toxicological and chemical analysis techniques to identify bioactive CECs remains challenging and laborious. In this study, we incorporate state-of-the-art identification approaches in EDA and propose a robust workflow for the high-throughput screening of CECs in environmental and human samples. Three different sample types were extracted and chemically analyzed using a single high-performance liquid chromatography HRMS method. Chemical features were annotated by suspect screening with several reference databases. Annotation quality was assessed using an automated scoring system. In parallel, the extracts were fractionated into 80 micro-fractions each covering a couple of seconds from the chromatogram run and tested for bioactivity in two bioassays. The EDA workflow prioritized and identified chemical features related to bioactive fractions with varying levels of confidence. Confidence levels were improved with the in silico software tools MetFrag and the retention time indices platform. The toxicological and chemical data quality was comparable between the use of single and multiple technical replicates. The proposed workflow incorporating EDA for feature prioritization in suspect and nontarget screening paves the way for the routine identification of CECs in a high-throughput manner.

## Abstract 2

A comprehensive workflow was developed that incorporates effect-directed analysis in suspect and nontarget screening for feature prioritization, allowing for the high-throughput identification of bioactive chemicals of emerging concern.

## 1 Introduction

The focus of chemical screening has shifted over the last decade from targeted analysis of a limited group of known compounds to new screening techniques to detect a broader spectrum of chemicals that are of emerging concern. Chromatography coupled to high-resolution mass spectrometry (HRMS) allows for the detection of several thousands of accurate masses (features) in a sample in a single measurement regardless of their origin and whether they are of concern for environmental or human health.^1^ Nontarget and suspect screening allows for the annotation of features; however, chemical identification remains challenging and laborious and requires confirmation with analytical standards. Consequently, prioritization steps are required to determine which chemical features warrant further identification.^1^ An experiment-driven prioritization approach is effect-directed analysis (EDA), which guides screening efforts specifically to those chemical features that exhibit a toxicological mechanism of action related to an adverse outcome. In EDA, a sample extract is fractionated and tested in in vitro or small-scale in vivo bioassays; only features related to bioactive fractions are considered for identification using HRMS data. EDA reduces the chemical complexity of the sample and facilitates the high-throughput screening of bioactive chemicals of emerging concern (CECs) in abiotic and biotic samples.

An increasing number of in vitro bioassays with different toxicological endpoints have been developed and validated for application in EDA studies.^2−5^ Together with the implementation of high-resolution fractionation, this allows for high-throughput toxicity testing of micro-fractions of a couple of seconds from a chromatographic run.^6^ In parallel, substantial progress has been made in the data processing of nontarget HRMS data such as the development of specialized commercial and open-source software tools that include peak picking algorithms^7−9^ and chemical libraries that allow suspect screening of large numbers of chemicals.^10−13^ To improve the identification of bioactive chemicals in EDA by HRMS screening, we aimed to incorporate novel processing techniques in our EDA workflow and investigate which experimental and data processing steps enhance chemical identification throughput and confidence.

The goal of this study was the comprehensive integration of state-of-the-art HRMS identification approaches with high-throughput fractionation and bioassays as a means of chemical feature prioritization in EDA. Here, we propose an EDA workflow for the prioritization of features and identification of bioactive compounds in multiple matrix types and we make suggestions to further enhance throughput for the rapid screening of environmental and human samples. For this, (1) wastewater treatment plant (WWTP) effluent spiked with antimicrobial agents, (2) the dust standard reference material (SRM) 2585, and (3) fetal calf serum (FCS) spiked with thyroid hormone (TH) system disrupting compounds were extracted and analyzed using HPLC-HRMS. Furthermore, high-resolution fractionation of the samples was performed using the FractioMate and the fractions were tested for their capacity to inhibit the bacterial growth of a sensitive*E. coli*clone (antibiotics assay)^14^ and for their capacity to compete with TH for binding to the distributor protein transthyretin (FITC-T4 TTR-binding assay).^15^ Finally, a comprehensive suspect screening approach, using a reference database (i.e., CECscreen), spectral libraries (i.e., MassBank of North America and EU MassBank), and in-house standard mixtures, was applied, and the annotation quality of annotated features was assessed. We determined the significance of technical replicates on the bioassay and identification results and assessed how EDA (as an experiment-driven prioritization approach) impacted feature reduction and the identification of (un)spiked compounds.

## 2 Materials and Methods

**Figure 1:**
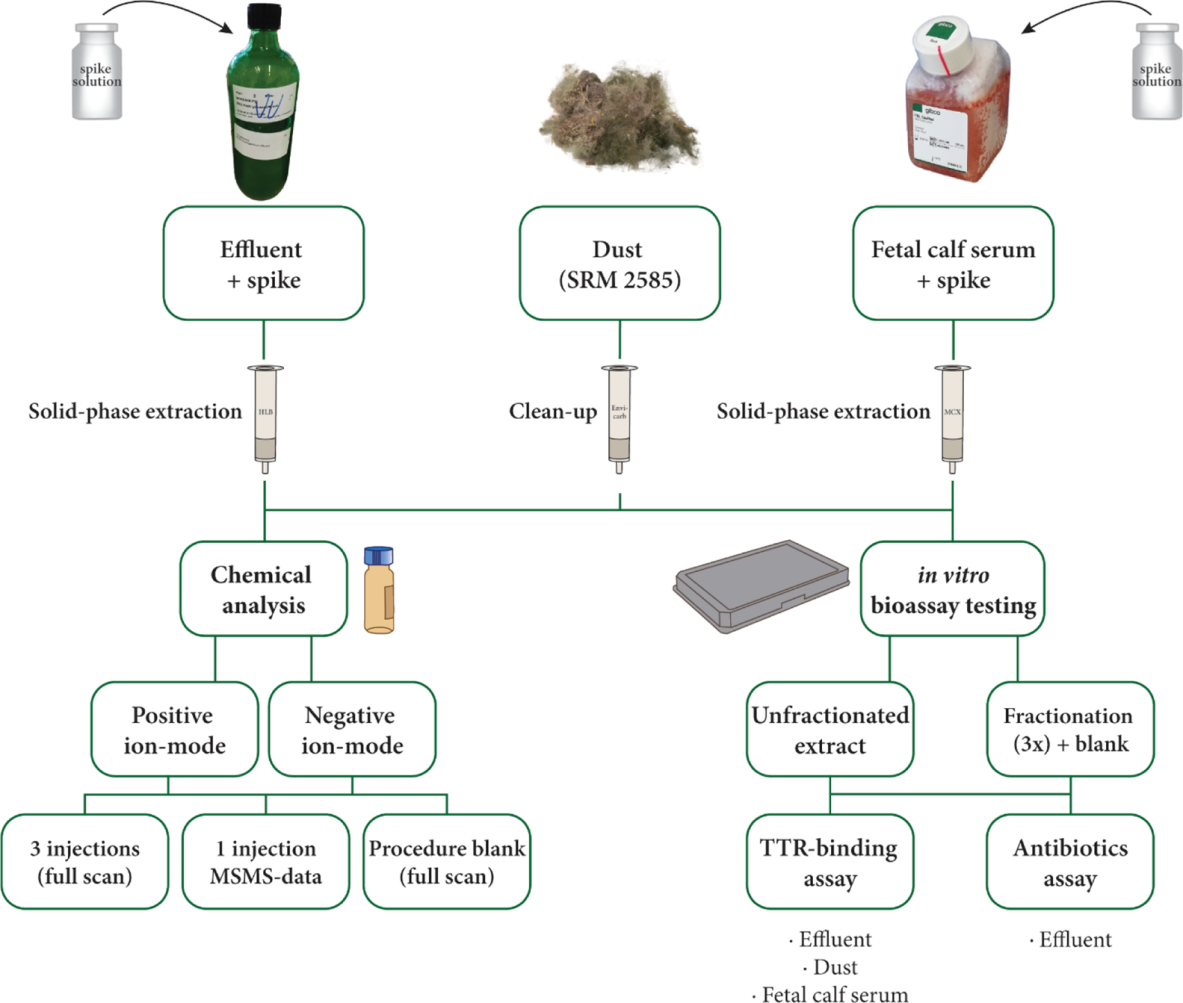
Experimental setup of the study. WWTP effluent spiked with antimicrobial agents, FCS spiked with TH system disrupting compounds, and dust SRM 2585 were extracted. Chemical analysis using QTOF-MS was performed on the extracts in the negative and positive ion modes. The samples were analyzed three times in full scan MS and once in data-dependent acquisition (DDA) MS/MS mode. In parallel, the extracts were fractionated. The unfractionated and fractionated extracts were tested in the TTR-binding assay (effluent, dust, and serum) and the antibiotics assay (effluent).

A schematic overview of the experimental setup of the study is provided in (Figure 1).

### 2.1 Spiking and Extraction of Samples

#### 2.1.1 Wastewater Treatment Plant Effluent

A 24 h composite effluent sample was obtained from the European Pollutant Release Transfer Register monitoring program 2019, location Kralingseveer, The Netherlands, which receives a small contribution of industrial wastewater (∼10%). EDTA was added to the 500 mL sample as a chelating agent (100 μM) to improve the extraction efficiency with solid-phase extraction (SPE), as fluoroquinolones and macrolide antibiotics may form complexes with metals or multivalent cations.^16^ The sample was filtrated through a Whatman GF/F filter (0.7 μm) and spiked with 0.5 μg of an isotope-labeled antibiotic standard solution of ciprofloxacin-d8 hydrochloride hydrate (Sigma-Aldrich, Zwijndrecht, The Netherlands), azithromycin-13Cd3, and clarithromycin-*N*-methyl-13Cd3 (Campro Scientific GmbH, Veenendaal, The Netherlands), giving a final environmentally realistic concentration of 1.0 μg/L each. The sample was acidified with formic acid (BioSolve, Valkenswaard, The Netherlands) to pH 3.0 and extracted with Oasis HLB cartridges (6 cc, 500 mg). The compounds were eluted with methanol (BioSolve, Valkenswaard, The Netherlands) (3 × 3 mL) and split into two equal parts. The extracts were evaporated until dryness at 40 °C under a gentle nitrogen flow, where one part was dissolved in 1 mL of 10% (v/v) methanol in Milli-Q [water purified on a Milli-Q Reference A+ purification system (Millipore, Bedford, MA)] and the other part in 50 μL of dimethyl sulfoxide (DMSO) (Acros, Geel, Belgium). A procedure blank (500 mL Milli-Q) was extracted in parallel and included in the chemical analysis and bioassay measurements.

#### 2.1.2 Dust

The SRM 2585 [National Institute of Standards And Technology (NIST), Gaithersburg (MD), USA] was selected as a representative dust sample for our study. This SRM contains a wide range of organic contaminants that may competitively bind to transthyretin (TTR)^17^ [e.g., perfluorinated alkylated substances (PFAS)]. An amount of 150 mg of SRM2585 was extracted according to the method utilized by Ouyang et al.^18^ In brief, ultrasonication of the sample was performed using acetonitrile (BioSolve, Valkenswaard, The Netherlands) and methanol, followed by a clean-up step. Envicarb SPE cartridges (Supelco, Zwijndrecht, The Netherlands) were activated with a mixture of methanol/acetonitrile (1:1, v/v), loaded with sample, and eluted with methanol. The extract was evaporated to almost dryness under a gentle nitrogen flow at room temperature and reconstituted in 600 μL of methanol/water (1:1, v/v). Subsequently, 40 μL of the final extract was transferred to a separate vial and evaporated to almost dryness and reconstituted in 40 μL of DMSO.

#### 2.1.3 Fetal Calf Serum

FCS was spiked with a mixture of the following seven TH system disrupting compounds: TBBPA, 2,4,6-TBP, 5-OH-BDE47, 6-OH-BDE47, 6-OH-BDE99, 4-OH-CB107, and 4-OH-CB187 (J.T. Baker, Deventer, The Netherlands). Nine milliliters of FCS was spiked with 6 μL of the spiking mixture in DMSO to reach a final concentration range of 0.015–0.115 μM of the seven compounds (Table S1). The binding potencies of the spiked compounds relative to T4 are included in Table S1, as determined by Hamers et al.^17^ The spiked FCS sample and a procedure blank of 9 mL of Milli-Q were extracted and cleaned up according to the method developed and validated by Simon et al.^19^ In short, plasma proteins were denatured with acidified 2-propanol (BioSolve, Valkenswaard, The Netherlands) and dissolved in a 2-propanol/water mixture. Subsequently, SPE with MCX cartridges was performed, followed by elution with 100% methanol. The extracts were evaporated under a gentle nitrogen flow at room temperature to approximately 300 μL of methanol, after which 300 μL of Milli-Q was added. Finally, for the unfractionated extract to be tested in the bioassay, 40 μL of the extracts was transferred to a separate vial and evaporated to almost dryness under a gentle nitrogen flow at room temperature and reconstituted in 40 μL of DMSO.

### 2.2 LC–MS(MS) Analysis and Fractionation

Sample extracts (20 μL) were injected with an Agilent 1290 Infinity HPLC system (Agilent Technologies, Amstelveen, The Netherlands) and analytes were separated on a BEH C18 column (Waters, 100 mm × 2.1 mm, 1.7 μm) set at 30 °C. Acetonitrile (ACN) (0.1% formic acid) and Milli-Q (0.1% formic acid) were used as solvents, and the flow rate was 500 μL/min. The gradient was increased linearly from 10 to 99% ACN (0.1% formic acid) in 18 min, where it was kept for 7.5 min. The column was equilibrated by returning to 10% ACN (0.1% formic acid) in the subsequent 0.5 min, where it was kept for 4 min. All samples were run in a consecutive sequence. The stability of retention times was assessed by injecting a standard—containing compounds that elute over the whole chromatogram—throughout the sequence. HRMS data were recorded on a Bruker Daltonics Compact II QTOF mass spectrometer (Bruker, Bremen, Germany). Electrospray ionization (ESI) was used to ionize compounds; full-scan (MS) and MS/MS scans were recorded in the positive and negative ion modes from 50 to 1300 *m*/*z* at spectra rates of 2 Hz (MS) and 5 Hz (MS/MS), respectively. Further details on the (source) settings of the instrument and data acquisition of (DDA) MS/MS data are provided in the Supporting Information (Tables S2–S4). Mass measurements were calibrated by injecting a tuning mix at the beginning of each sample injection.

Fractionation was performed with a FractioMate fraction collector (SPARKHolland & VU, Emmen & Amsterdam, the Netherlands) using the same HPLC conditions as described above.^6^ The enrichment factor in the fractionated plates for the TTR-binding assay was 10 times higher and for the antibiotics assay 2.5 times higher compared with the highest concentration tested of the unfractionated samples. Post-column, the eluent was fractionated into 80 wells of a 96-well plate of the respective bioassay (positions A3-H12) that were filled with 10 μL of keeper solvent (10% DMSO in Milli-Q). Each fraction corresponded to a 13.5 s interval of the LC-run. After fraction collection, the well plates were dried in a CentriVap concentrator (Labconco, Kansas City, United States) under vacuum for approximately 4 h at 25 °C.

### 2.3 In Vitro Bioassays

The TTR-binding assay and the antibiotics assay were selected based on their relevance for human and environmental health.^17,20^ Furthermore, the bioassays were assigned to matrices for which they have been validated. Another important attribute for the selection of assays was the suitability for use in a 96-wells format, permitting micro-fractionation. The TTR-binding assay has been used for serum,^19^ dust,^18^ and water samples^21^ previously, whereas the antibiotics assay is only validated for water samples.^14^

#### 2.3.1 Antibiotics Assay

This cell-based assay monitors the bacterial growth of*E. coli*FhuAT, a Gram-negative strain that is susceptible to a wide range of antibiotics as it carries an open variant of an outer membrane protein channel combined with an inactivated multidrug efflux transport system.^14^ Growth-inhibiting effects were determined in the logarithmic growth phase of the cells. The antibiotics assay was performed according to Jonkers et al.^14^ as described in Section S1 of the Supporting Information.

#### 2.3.2 TTR-Binding Assay

The TTR-binding assay measures the competitive binding of chemicals to transthyretin (TTR) in the presence of a fluorescent conjugate of T4 and fluorescein 5-isothiocyanate (FITC). In short, this FITC-T4 conjugate shows high fluorescence when its T4-group is bound to TTR. When a competitive binder of TTR is present, however, the FITC-T4 is repressed from the binding site of TTR, resulting in a lower fluorescent signal due to intermolecular quenching of the fluorescein group. The assay was performed according to Hamers et al.^17^ with some modifications, as described in detail in Section S1 of the Supporting Information.

#### 2.3.3 Bioassay Measurements and Hit-Selection

Fractionated extracts were tested in triplicate; the fractionated procedure blanks were tested in a single measurement. The methods of testing the fractionated samples in the bioassays are described in detail in Section S1. Bioactive fractions were distinguished from background noise by comparing the activity of the individual fraction to a hit-threshold. The hit-threshold for the antibiotics assay was set at the response of the procedure blank minus 3× the standard deviation of the procedure blank response of all fractions. The hit-threshold for the TTR-binding assay was set at 20% fluorescence inhibition compared to the control, the approximate response level where the linear part of the dose–response curve starts for T4 (Figure S2). Unfractionated extracts were tested in eight dilutions (*n* = 2) for the TTR-binding assay and nine dilutions (*n* = 3) for the antibiotics assay.

### 2.4 Data-Processing Strategy

MetaboScape 4.0 (Bruker, Bremen, Germany) was used for the ion deconvolution of MS(MS)-data and subsequent peak identification. The T-ReX 3D processing workflow (detailed settings can be found in Table S5) was applied separately for each matrix type (*n* = 3), injection (*n* = 3), and ion mode (*n* = 2), resulting in 18 feature tables. The software performed an automated mass calibration and de-isotoping algorithm, of which the retention times of the resulting features were aligned with a LOESS-based alignment algorithm.^9^

### 2.5 Annotation Workflow

The extracted features were annotated in MetaboScape by matching the measured accurate mass (mass deviation ≤10 ppm), retention time (RT deviation ≤0.2 min), isotopic peak pattern fit (mSigma ≤100; a measure describing the relative mean square difference of a measured and theoretical isotopic pattern),^22^ and MS/MS-score (≥600) to that of a suspect. The suspect lists that were hierarchically applied are listed in Table 1. The annotation quality of each annotation was assessed with the total annotation quality code (TAQ-code). This newly developed code (Figure S1) includes not only the mass accuracy, retention time, isotopic pattern fit, and MS/MS spectra but also the presence of MS/MS data and whether the feature met the inclusion criteria for feature intensity (sample ≥3× blank) and coefficient of variance between multiple injections (≤20%). The cutoff values for the TAQ-code were based on the specifications of the applied HRMS instrument for accurate mass and isotopic pattern fit. The retention time deviation was based on the peak width resulting from the applied LC-method (0.1 min). The applied TAQ-code was further used to assign an identification confidence level to each annotation following the levels proposed by Schymanski et al.,^23^ that is, a confirmed structure by a reference standard (level 1), a probable structure by a library spectrum match (level 2a) or by diagnostic evidence (level 2b), a tentative candidate (level 3), an unequivocal molecular formula (level 4), and an exact mass (*m*/*z*, level 5). Level 4 annotations were divided into two categories: features with and without recorded MS/MS spectra. Throughout the paper, level 4 annotations for which MS/MS data were recorded are referred to as level 4* annotations, unless stated otherwise. The relation between the TAQ-code and the identification levels proposed by Schymanski et al.^23^ is explained in the Supporting Information (Table S6). In case features had multiple annotations from different suspect lists, the annotation with the highest identification confidence level was selected as the primary candidate.

**Table 1:** In Total, Six Suspect Lists Were Applied on the Feature Tables to Annotate Extracted Features^a^

list name	type	identification parameters	number of compounds	reference
MassBank of North America	SL	exact mass, mSigma,MS/MS	17,747 (72,439 spectra)^e^	(10)
EU MassBank	SL		19,791 (88,168 spectra)^e^	(11)
CECscreen	AL	exact mass, mSigma	70,397^b^	Meijer et al.^24,25^
MCS1^d^	AL+	exact mass, RT, mSigma, MS/MS	131 (99^c^)	
MCS2^d^	AL+		409 (358^c^)	
antibiotics^d^	AL+		15	

a SL = spectral library, AL = analyte suspect list, AL+ = standards, and MCS = multicomponent standard.

b CECscreen without simulated metabolites.

c The number of compounds with a recorded retention time.

d In-house mixtures of standards.

e The number of spectra was not exclusively obtained with ESI.

#### 2.5.1 Increasing Annotation Confidence Levels

The identification confidence level of annotated features (especially level 4* and 4 annotations) can be improved with computational tools by for instance estimating the retention behavior of the annotated candidate and by matching in silico fragmentation data to recorded MS/MS spectra. Two of these tools, the retention time indices (RTI) platform^26^ (rti.chem.uoa.gr, accessed March 2021) and MetFrag,^27,28^ were assessed on their effectiveness to distinguish between isomers from CECscreen annotations. The RTI platform, a QSAR model, predicts retention times based on the structure (SMILES) of the suspect and compares that to the measured retention time.^26^ The OTrAMS model was selected to estimate the uncertainty of the RTI prediction. The output is divided into four applicability domain boxes with varying levels of reliability.^29^ Boxes 1 and 2 include structures with matching experimental and predicted retention times (although the error is larger in box 2). Boxes 3 and 4 include structures without matching retention times. Details on the applicability domain boxes are described by Aalizadeh et al.^29^

## 3 Results and Discussion

### 3.1 HRMS Identification of Chemical Features at Different Identification Confidence Levels

Multiple features were detected and annotated for each matrix type at different annotation confidence levels. A summary of the results are provided in the Supporting Information (Tables S7–S9). The data tables were processed separately for the triplicate, duplicate, and single sample injections and showed a comparable number of annotations. The majority of level 1 and 2b annotations were pharmaceuticals and pesticides, whereas the spectral library matches (level 2a) were mainly endogenous compounds and phthalates for dust and endogenous compounds for serum. Level 4* and 4 annotations, which are based on exact mass and isotopic pattern fit, were primarily CECscreen annotations. As the CECscreen database is large (>70,000 compounds), one annotation may reflect multiple isomers that cannot be distinguished without the use of additional tools or information. An overview of all annotations (including isomers for CECscreen annotations) can be found on Zenodo (DOI:10.5281/zenodo.5657052).

### 3.2 Bioassay Response to the Unfractionated and Fractionated Extract

**Figure 2:**
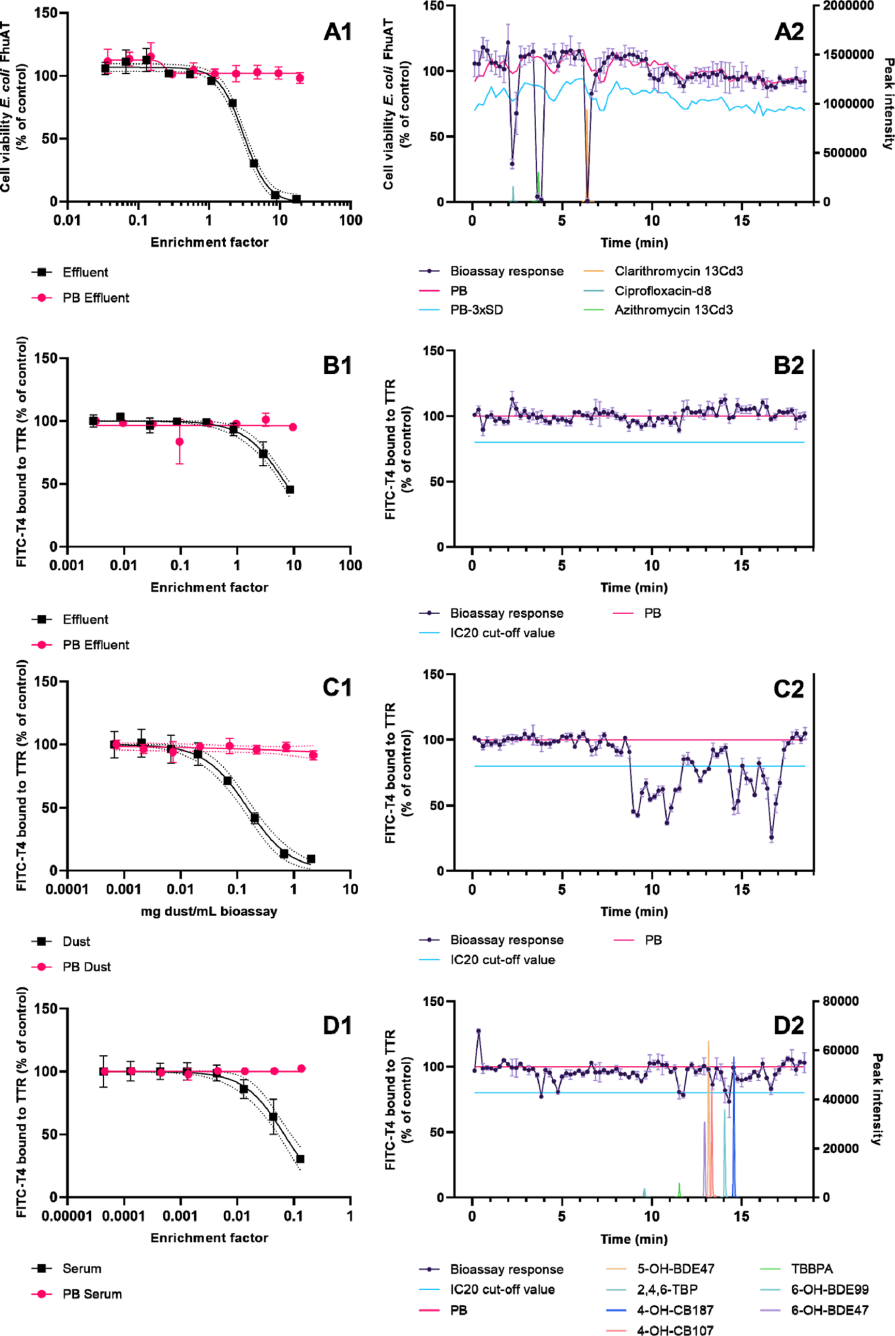
Bioassay responses to unfractionated (A1–D1) and fractionated (80) extracts (A2–D2) of spiked wastewater effluent (A: antibiotic bioassay response; B: TTR-binding assay response), dust SRM2585 (C: TTR-binding assay response), and spiked FCS (D: TTR-binding assay response). PB = procedure blank. Error bars represent standard deviations of the technical replicates. The MS-peak intensities of compounds that were spiked are plotted on the right *Y*-axis for the relevant matrix-types (A2,D2).

The unfractionated and fractionated extracts were tested in the bioassays, where the TTR-binding assay was tested for each matrix type and the antibiotics assay for effluent only. All of the unfractionated extracts showed concentration-dependent activity in the bioassays (Figure 2). Concerning the fractionated extracts, each bioassay data point is plotted in the middle of the fraction time in Figure 2(A2–D2) (i.e., fraction 1 runs from 0 to 13.5 s; the data point is plotted at 6.75 s), while the MS-chromatograms describe the peak intensities that were recorded for the spiked compounds at that retention time. In the fractionated effluent extract, five bioactive fractions were identified with the antibiotics assay which can be grouped into three active regions (Figure 2, A2). No bioactive fractions were identified with the TTR-binding assay for this matrix (Figure 2, B2), although the unfractionated extract showed TTR-binding activity at the two highest tested enrichment factors (Figure 2, B1). The response in the unfractionated extract may be explained by the additive effects of multiple individual compounds. After separation (Figure 2, B2), the individual concentrations or potencies may be too low for a measurable effect.^30^ The fractionated dust extract showed 28 bioactive fractions in the TTR-binding assay (Figure 2, C2) and the fractionated serum extract contained six bioactive fractions (Figure 2, D2). A response was observed in each bioassay of the unfractionated extract at the highest tested enrichment factor (Figure 2). The enrichment factors were 2.5–10 times higher in the fractionated plates and therefore bioactivity was expected in the fractionated samples assuming the activity was caused by a limited number of compounds.

### 3.3 Identification of Features in Bioactive Fractions

**Figure 3:**
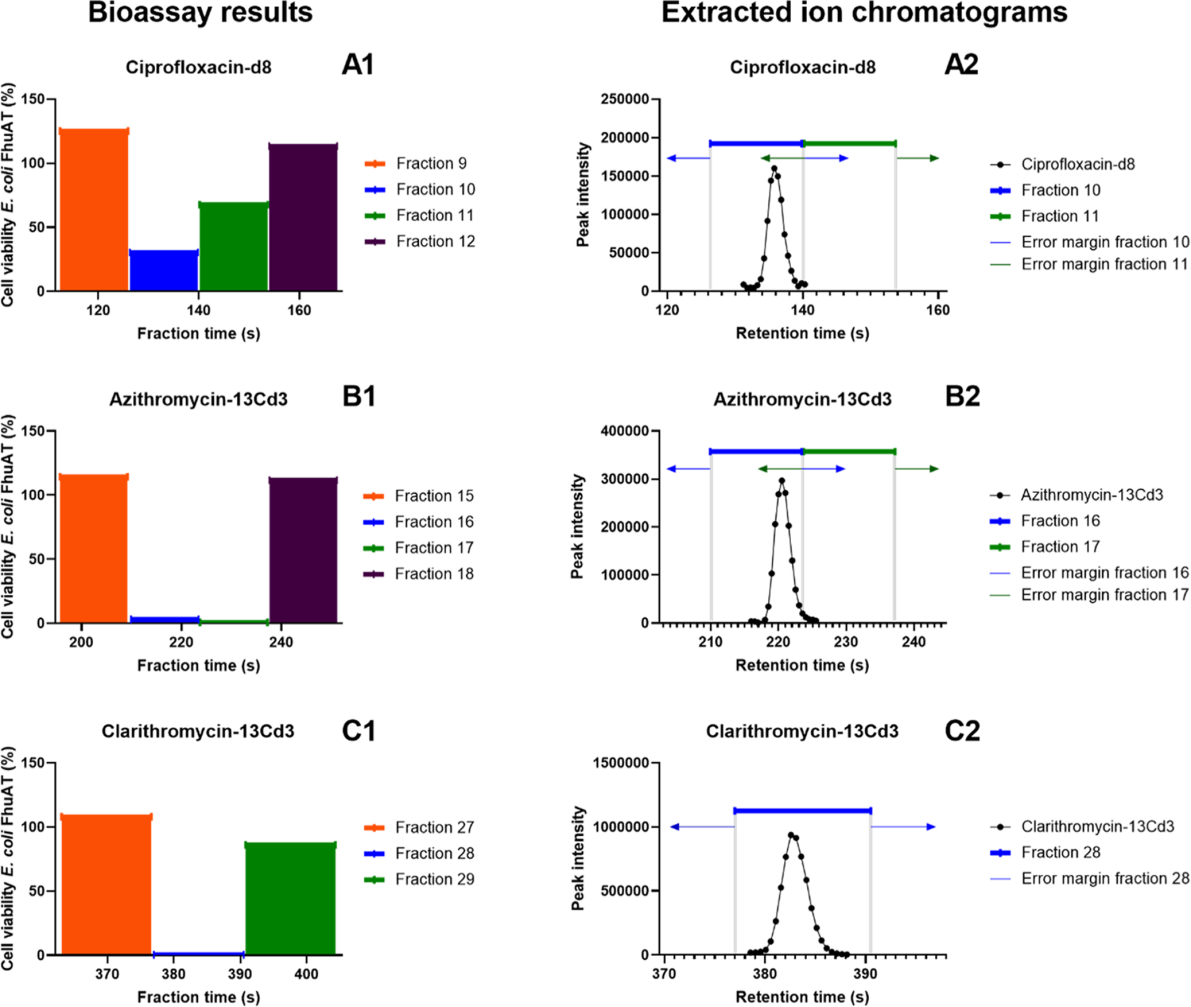
Alignment of the bioassay result and the MS-chromatograms of spiked compounds, exemplified by the fractionated effluent extract as measured in the antibiotics assay. The left-sided figures represent the bioassay results for the three active regions (A1,B1,C1) on the fractionated antibiotics assay plate and the adjacent fractions. Here, the cell viability of*E. coli*FhuAT is plotted against the fraction time. The right-sided figures represent the extracted ion chromatograms of the antibiotics spiked to effluent and are plotted as signal intensity against retention time. Here, each data point represents a full scan MS-measurement for the exact mass of ciprofloxacin-d8 (A2), azithromycin-13Cd3 (B2), and clarithromycin-13Cd3 (C2) (*m*/*z* of [M + H]^+^ ± 5 mDa). Furthermore, the retention time windows of the corresponding fractions—as recorded by the FractioMate—are highlighted. The error margins of 6.5 s are presented as arrows.

Annotated features were selected from retention time windows that corresponded to the bioactive fractions. The alignment of the bioassay result and MS-chromatogram is visualized in Figure 3. The bioassay results show that there is a difference in the retention times of the fractionated plates and the MS-measurements, that is, A1 and B1 show antimicrobial activity in two subsequent fractions, whereas the activity was expected to occur in a single fraction based on the extracted ion chromatograms of the spiked compounds ciprofloxacin-d8 (A2) and azithromycin-13Cd3 (B2). On the other hand, clarithromycin-13Cd3 indeed eluted into a single fraction (C1) as was expected according to the MS chromatogram (C2), meaning that the difference in retention times between the fractionated plate and the MS-chromatogram was at most a few seconds. No retention time shifts were observed in the standard mixture that was injected throughout the sequence. Therefore, this difference is likely caused by a mechanical difference between the MS-instrument and the FractioMate. An error margin of 6.5 s (approximately half the fraction length of 13.5 s) was applied to the retention time windows to correct for possible errors in the alignment of the bioassay and MS chromatogram. Consequently, the length of each retention time window was 26.5 s per bioactive fraction. Table 2 describes for a selection of active fractions the distribution in the number of annotated features processed for single injections, separated according to identification strength and matrix type. These results processed for multiple injections are presented in Table S10. The peak shapes of features annotated at a high identification confidence level (levels 1, 2a, and 2b) were manually assessed. In total, 97% of the peak shapes were classified as acceptable (Gaussian peak shape).

**Table 2:** Selection of Bioactive Fractions for Each Matrix Type Describing the Combined Number of Features Measured in Positive and Negative Ion Modes That Were Related to the Fractions for Single Injections^a^

matrix	fraction	*n*	level 1 (n)	level 2a (n)	level 2b (n)	level 3 (n)	level 4*^b^ (n)	level 4 (n)	level 5 (n)
effluent	10	569	1*H*-benzotriazole acid	2-benzothiazolesulfonic acid	ciprofloxacin-d8	0	20 (145)	52 (400)	488
			2,5-dimethylbenzenesulfonic acid	2,5-dimethylbenzenesulfonic acid					
			amantadine	3,4-methylenedioxy-*N*-methylamphetamine (MDMA)					
			lidocaine	leucyl leucine					
	11	521	lidocaine	2,5-dimethylbenzenesulfonic acid	1,3-diphenylguanidine	1	16 (85)	47 (309)	453
					ciprofloxacin-d8				
	16	434		triethyl phosphate	azithromycin	2	20 (22)	58 (287)	351
					azithromycin-13Cd3				
	17	439	bisoprolol A	S,R-noscapine	azithromycin	21	56 (111)	354 (471)	354
			clozapine	triethyl phosphate	azithromycin-13Cd3				
	28	192	DEET (*N*,*N*-diethyl-3-methylbenzamide)	amitriptyline	clarithromycin-*N*-2 methyl-13Cd3	2	12 (66)	53 (493)	115
			losartan	candesartan	clarithromycin				
				cetirizine					
				climbazole					
				methadone					
				*N*-desmethyl clarithromycin					
dust	39	540		*N*-octyl-2-pyrrolidone		1	22 (310)	86 (1515)	430
	40	370		lauryl diethanolamide		1	16 (237)	85 (1803)	267
	47	383	diazinon	dibutyl phthalate		0	13 (210)	70 (1295)	297
				*N*-dodecanoyl-*N*-methylglycine					
	63	333		anthranilic acid		1	13 (223)	57 (632)	261
	64	381		anthranilic acid		0	11 (172)	70 (1116)	298
				oleyl sarcosine					
	72	313				1	12 (122)	52 (1018)	248
serum	17	179		4-hydrobenzoic acid	bisphenol S	1	23 (292)	58 (673)	93
				azelaic acid					
				indoleacetic acid					
	21	208		decanedioic acid		1	34 (293)	93 (1441)	78
				hippuric acid					
	50	223		fipronil sulfone		0	12 (70)	60 (485)	148
				LPC 16:0					
				LPC 18:1					
	51	357		LPC 16:0		3	13 (105)	58 (331)	281
				LPC 18:1					
	61	206				3	10 (34)	46 (313)	147
	62	207				2	12 (39)	46 (327)	147

a Features are ordered according to the identification confidence level of which level 1 and 2 annotations are shown by name. The total number of possible isomers identified through CECscreen is shown between parentheses.

b Level 4-features for which MS/MS spectra were recorded.

#### 3.3.1 Identified Features in Bioactive Fractions of Effluent

The spiked antibiotics were identified in the bioactive fractions at identification confidence level 2b, meaning the annotations occurred on exact mass (<5 ppm), retention time (<0.1 min), and isotopic pattern fit (<25 mSigma) with an in-house suspect list (Table 1, antibiotics). This suspect list did not contain MS/MS spectra from isotope-labeled antibiotics. As such, the spiked compounds were not identified at level 1. In addition to the spiked compounds, high-level identifications in bioactive fractions (levels 1, 2a, and 2b)—with possible antimicrobial effects—were 2-benzothiazolesulfonic acid (a fungicide or additive used in the production of rubber, fraction 10),^31^ 1.3-diphenylguanidine (a catalyzer used in rubber materials^32^ and classified as toxic to aquatic life with long-lasting effects,^33^ fraction 11), azithromycin and sulfamethoxazole (antibiotics, fraction 16), azithromycin (antibiotic, fraction 17), and DEET (*N*,*N*-diethyl-3-methylbenzamide), clarithromycin (antibiotic), and *N*-desmethyl clarithromycin (antibiotic metabolite) in fraction 18. The antimicrobial effects of the isotope-labeled antibiotics are indistinguishable from the effects of the native antibiotics, as they elute in the same fraction. No other antibiotics were identified with the suspect lists that were applied, which agrees with the absence of bioactivity in other fractions. The adequacy of applying a 26.5 s retention time window in the feature selection to include a possible bioactive feature is shown by the identification of azithromycin in both fractions 16 and 17.

The TTR-binding assay identified no active fractions in the fractionated effluent sample, even though perfluorooctanoic acid (PFOA), which can bind to TTR,^17^ was identified at level 2b (RT = 8.5 min). The PFOA concentration in the fraction was probably too low for a significant response, as PFOA is not very potent in the assay [IC_50_-value = 1.1 μM (455 μg/L)].^17^ The enrichment factor for the fractionated effluent in the TTR-binding assay was 12.5. As such, the PFOA concentration in the effluent should have been ≥36.4 μg/L for a 50% inhibition response in the TTR-binding assay. For comparison, this concentration exceeds the maximum PFOA concentration of 60 ng/L in German WWTP effluent^34^ by almost 3 orders of magnitude.

#### 3.3.2 Identified Features in Bioactive Fractions of House Dust

SRM 2585 contains organic contaminants for which certified mass fraction values (NIST-certified values) have been determined, including tetrabromobisphenol-A (TBBPA) and PFAS,^35^ that may competitively bind to transthyretin (TTR).^17^ Other compound classes present in SRM2585 are, for example, polycyclic aromatic hydrocarbons, chlorinated pesticides, and polycyclic musks.^35^ The CECscreen database (level 4* and 4 annotations) contains the majority (92.5%) of the compounds with a NIST-certified value in SRM 2585,^35^ but only four musk-type compounds, two flame retardants, and a PFAA (PFNA) were annotated over the whole chromatogram. Most of the compounds for which NIST-certified values are reported were measured using GC/MS, which might explain the low number of tentatively identified compounds using LC/MS with ESI.^35^ Of the annotated NIST-certified compounds, only PFNA has known TTR-binding activity^17,36^ and indeed eluted in a fraction that was bioactive (fraction 39). The effect measured in this fraction cannot be explained by the presence of PFNA alone, as the concentration was ≤5 nM (estimated from the SRM2585 NIST certificate, assuming 100% extraction recovery) and its IC_50_-value is 1.1 μM.^17^ Of the remaining annotated NIST-certified compounds, the flame retardant tributyl phosphate and the synthetic musks ADBI and AHMI eluted in wells that showed bioactivity. The TTR-activity of these compounds was determined, but none of the compounds showed TTR-binding activity up to a test concentration of 150 μM.

All high-level identifications (levels 1, 2a, and 2b) in the sample with anticipated or known effects in the TTR-binding assay, such as nonafluorobutane-1-sulfonic acid (PFBS), perfluoroheptanoic acid (PFHPA), or 6:2 fluorotelomer sulfonic acid (6:2 FTSA), eluted in fractions without activity. Most probably, their concentrations were too low to measure an effect. Diazinon, an organophosphate insecticide, was identified at level 1 in active fraction 47. However, no TTR-binding activity was observed for diazinon in a previous study using the radioligand TTR-binding assay (unpublished results). Table 2 shows that a large number of candidate features remain that could be investigated further to identify the other causative compounds.

#### 3.3.3 Identified Features in Bioactive Fractions of Serum

The spiked serum compounds remained unidentified in the bioactive fractions despite their presence, as confirmed by manual inspection of the MS data (Figure 2, D2). We found that the peak deconvolution algorithm had defined the incorrect *m*/*z*-value as monoisotopic mass, most probably as a result of the complex isotope profile of multihalogenated compounds. All of the compounds spiked to serum were multihalogenated. The open-source software HaloSeeker^37^ is an alternative for the identification of multihalogenated compounds. Its use has been successfully demonstrated with marine sediment and human milk HRMS data.^37,38^ This software is able to filter polyhalogenated signals from HRMS data and assign chemical formulas.^37^ The identification of multihalogenated compounds will be addressed in future work. This addresses a drawback of suspect and nontarget screening, which is relying on complex software tools to extract features (*m*/*z*-values) from HRMS data. Omissions and shortcomings that may occur are difficult to track down retrospectively. Also, these software tools use unique algorithms for this purpose that can lead to different results when analyzing the same data.^39^ Consequently, transparency of the applied workflow and follow-up data processing steps need to be carefully addressed in nontarget screening studies, so the reproducibility and comparability among studies can be assessed.^39^ Thorough QA/QC analyses of spiked samples should be included in EDA studies to evaluate the method performance. None of the bioactive fractions in serum had annotations at level 1. Spectral library matches (level 2a) included endogenous acids (e.g., 4-hydrobenzoic acid, fraction 17; decanedioic acid, fraction 21), lipids (e.g., LPC 16:0, fraction 51), but also the pesticide fipronil sulfone (fraction 50). In fraction 17, bisphenol S was identified at level 2b. Fipronil sulfone and bisphenol S were tested in the TTR-binding assay. Both compounds showed a concentration-dependent effect in the assay with IC50 values of 14 and 73 μM, respectively (Figure S3). The chemical identities of fipronil sulfone and bisphenol S were confirmed by comparing the chemical analysis (negative ion mode) of the serum sample and the corresponding analytical standards, resulting in matching retention times and matching fragmentation patterns of the MS/MS spectra (Figures S4 and S5). It is remarkable that bioactive compounds such as fipronil sulfone and bisphenol S are present in FCS, a product that is widely used as a growth supplement for the in vitro cultivation of cells.

Fractions 17 and 21 (RTs of 3.8 and 4.8 min, respectively), showed activity not introduced by the spiked compounds (Figure 2, D2). By manually inspecting the chromatogram, a high-intensity peak was found at the RT of 3.8 min with a distinct isotopic pattern of a structure with two bromine atoms. The corresponding MS/MS spectrum was analyzed with the MetFrag web tool using PubChem as a compound database. All major fragments were matched to (4,5-dibromo-2-hydroxy-3,6-dimethylphenyl) hydrogen carbonate, a compound structurally similar to the metabolites of polybrominated diphenyl ether flame retardants spiked to the serum (Figure S6). This compound, although not confirmed by a standard, may be a degradation product of one of the spiked brominated flame retardant that can still competitively bind with TTR.

### 3.4 Impact of Multiple Injections on Peak Picking and Annotations

The number of injections had little impact on the number of features in the bioactive fractions, especially on level 1, 2, and 3 annotations (Table S10). Except for fractions 10 and 11 of the effluent sample, the total number of annotations increased slightly with more injections. This may be explained by the recursive feature extraction that was applied, where features that had not been selected in prior analyses were picked recursively based on the presence of that feature in other analyses. In such cases, the selection criteria to extract a feature (such as peak intensity or peak length, Table S5) were too strict to be included in the primary pass and less stringent criteria were applied to extract that feature.^9^ The numbers of level 4* annotations (level 4-features with MS/MS spectra) are in most cases not affected by this tool, as the DDA-mode of the QTOF requires a relatively high signal of precursor ions to be selected for fragmentation. The difference in the total number of annotations between injections was mainly driven by annotations with identification confidence levels 4 and 5.

The main advantage of multiple injections is that the data quality may improve. For example, the spectrum of a compound of interest may be of better quality in a second or third injection, which will mainly affect the ability of the software to distinguish a correct isotopic pattern. This will subsequently affect the identification strength of a feature. Also, the data processed as a single injection may contain more instrumental noise.^40^ Furthermore, recursive feature extraction, which is possible when injecting multiple technical replicates, results in the detection of slightly more chemical features. Considering the replicability of the applied bioassays (Figure 2) and the generally higher sensitivity of the HRMS instrumentation compared with the bioassays, it is likely that the intensity of the chemical feature responsible for the bioactivity is high, should it ionize with ESI. Especially in EDA studies, the marginal increase in the number of low-intensity features does not outweigh the gain in the reduction of time and resources using a single injection. It might be more beneficial to spend these resources to increase the sample throughput instead.

### 3.5 Increasing the Identification Confidence

Level 4* annotations may be improved to level 3 by estimating their retention behavior and by matching measured fragments to in silico predicted fragments. To illustrate this, one bioactive fraction was randomly selected from each matrix type. From each fraction, a level 4* CECscreen annotation (including isomers) was randomly selected in both positive and negative ion modes and processed with the RTI platform and MetFrag (Table 3). Information on the quality of the RTI platform calibration (comparing the retention behavior of a set of reference compounds of the applied LC-method with that of the RTI system) is presented in Figure S7. The RTI platform reduced the number of candidates (possible isomers, box 1) to be considered for further elucidation by 63% (±SD 25%) on average. Candidates from box 4 (outliers)^29^ were excluded from this calculation. It should be noted that the RTI platform is unable to accurately estimate the retention behavior of the candidates in box 4. These compounds could be reconsidered when the identity of box 1 candidates cannot be confirmed. Also, compounds with similar structures are more difficult to differentiate than dissimilar structures.

**Table 3:** Selected Chemical Features with CECscreen Annotations at Level 4* in Bioactive Wells for all Measured Matrices Processed with the RTI-Tool Including the Resulting Numbers of Isomers in Each Domain Box^a^

matrix	ion mode	feature #	RT (min)	*m*/*z*	isomers (n)	box 1 (n)	box 2 (n)	box 3 (n)	box 4 (n)
effluent (F10)^b^	(+)	1522	2.03	250.1808	15	3	4	1	7
	(−)	2701	2.43	199.0429	10	0	5	4	1
dust (F39)^c^	(+)	3837	8.86	186.1854	8	6	2	0	0
	(−)	6602	8.90	311.1658	10^d^	4	3	0	1
serum (F51)^c^	(+)	3367	11.87	303.2320	22	9	12	0	1
	(−)	4255	11.89	313.2373	18	4	14	0	0

a For box 1 predictions, the experimental and predicted retention times are accepted. The bioactive fraction for which the feature selection was done is depicted in between parenthesis below the matrix.

b Bioactive fraction in the antibiotics assay.

c Bioactive fraction in the TTR-binding assay.

d The RTI platform was unable to predict a retention time for two isomers.

MetFrag was applied on a selection of structures with accepted experimental and predicted retention times (box 1) to determine whether the remaining structures could be differentiated further with predicted fragmentation patterns, thereby increasing the identification confidence to level 3. Feature number 1522 was selected from effluent, 3837 from dust, and 4255 from serum. Masses with a relative abundance higher than 1% of the base peak in the MS/MS spectra of the selected features were extracted from MetaboScape and imported in the MetFrag Web tool. Predicted fragmentation patterns of the compounds in box 1 were compared to the measured MS/MS spectra using a relative mass deviation of 5 ppm to match the generated fragments against the measured *m*/*z* values. The resulting fragment predictions were compared to each other and weighed together with the accuracy of the retention time prediction to select the most likely candidate. Detailed information on the exercise including the candidates, predicted retention times, and fragment matches are provided in the Supporting Information (Table S11 and Figure S8). Feature number 1522 (effluent) included three candidates in box 1, namely, *N*-desmethyltramadol, *O*-desmethyltramadol, and procinolol with comparable predicted retention times. MetFrag was unable to match fragments for the first two candidates. For procinolol, the two most intense fragments could be matched with the predicted fragments (Figure S8). The remaining candidates with matching fragments still require manual interpretation of the spectra. For instance, in the case of procinolol, the in silico predicted fragments (such as cleavage of the bond within the benzene ring) are unlikely to occur in MS/MS. Similar exercises to improve annotation levels are demonstrated for feature numbers 3837 (dust) and 4255 (serum) in Figure S8.

### 3.6 Study Limitations and Recent Advancements

Despite the comprehensive annotation efforts, the majority of the chemical features remained unannotated because they were not present in one of the applied reference databases. This highlights a drawback of the suspect screening approach: the annotation process is guided by the compounds that are included in the applied reference databases. Therefore, reference databases should be selected with care and include metabolites and transformation products. Then, an annotation can be used as a prioritization step toward confirming the identity and bioactivity of that compound. Both unannotated and annotated features related to bioactive fractions can be prioritized further on signal intensity, peak shape, the presence of MS/MS data, or on the frequency by which a feature occurs in the analyzed samples.

The identification of unknown unknowns (compounds yet to be identified and not present in databases or the literature) is more difficult and requires additional means, for example, by determining elemental compositions combined with expert judgment of fragmentation patterns. A strategy to identify unknown active metabolites or degradation products from known (active) compounds includes predicted transformation products^41^ or reaction-based transformation experiments.^42^

In nontarget and suspect screening studies, MS/MS data play a key role in obtaining higher confidence identifications.^13^ A spectral library match of a distinct fragmentation pattern with that of a database provides a strong indication for the structure of the annotated compound. Therefore, there are an increasing number of initiatives aimed at organizing large collections of tandem mass spectral data of analyzed standards.^10,11,13^ Due to the applied DDA-acquisition approach in this study, which hierarchically selects precursors on signal intensity, fragmentation data were not recorded for all features that might be of interest (especially those that were of low intensity). Consequently, additional injections of the sample may be required to obtain the fragmentation data for specific features. A recent study incorporated, among other triggers, structural alerts to improve the collection of MS/MS data of potentially toxic compounds.^43^ As opposed to a hierarchical selection of precursors with high sensitivity, this approach triggers MS/MS events based on the detection of suspect list entries in the full scan. As a result, the probability of fragmenting a feature of interest is increased, especially considering biological samples where the highest intensity peaks are usually associated with endogenous compounds. A more comprehensive approach includes data-independent acquisition and more specifically SWATH-MS.^44^ In this approach all features within a certain precursor mass range are fragmented, which results in highly complex MS/MS spectra. Consequently, deconvolution of the MS/MS signals is more challenging and requires more elaborate deconvolution algorithms.^45^ Another recent advancement to obtain higher confidence identifications in suspect and nontarget screening studies for both environmental and human health is the use of ion-mobility by obtaining collision cross-sectional values.^46,47^ Although the incorporation of these techniques depends on the capabilities of the applied acquisition instrument and software, they may contribute to the throughput of sample screening (through efficient data acquisition) and to identifying CECs.

To assist in the prioritization of annotated features and in the automatic assignment of identification confidence levels, we have developed the TAQ-code. In addition, the transparency of the TAQ-code provides information on the individual parameters underlying the accuracy of the annotation, allowing QA/QC evaluation. The cutoff values for the TAQ-code were based on the specifications of the applied HRMS instrument and retention time characteristics. It is recommended to adjust the cutoff values of the TAQ-code to the resolution of the applied instrument and chromatographic performance (peak width and stability) if necessary.

In this study, separate injections were performed for fractionation and chemical analysis to minimize RT deviations. A post-column flow splitter device is often used in EDA studies, where one part of the split is directed toward the MS and the other to the fractionation device. The advantage of this approach is an enhancement in sample throughput.^48^ On the other hand, this approach affects chromatographic peak shapes and causes retention time shifts between the MS and bioassay chromatograms (Figure S9) that require correction.

### 3.7 Measurement Strategy and Suggested Workflow

For years, identifying toxicants in a high-throughput manner has been one of the bottlenecks of EDA. Our data show that a single full scan measurement combined with an MS/MS measurement, in positive and negative ion modes, give comparable results to those obtained with multiple technical replicates. Also, the antibiotics assay and TTR-binding assay were reproducible with standard deviations <20% (of normalized data) between technical replicates of the fractionated plates. Consequently, the use of single instead of multi-injections for full scan MS and bioassay sample measurements can further increase the throughput in sample screening, without compromising on data quality. The effectiveness of the workflow was demonstrated by the identification of the spiked antibiotics in the effluent sample. Furthermore, two novel TTR-binding compounds—fipronil sulfone and bisphenol S—were identified and confirmed in FCS. This workflow facilitates rapid screening and prioritization of features related to bioactive fractions, but confirmation of annotated compounds is still required which can be a laborious process. Also, unannotated features that were selected as a result of the workflow may require further investigation applying nontarget screening techniques.

**Figure 4:**
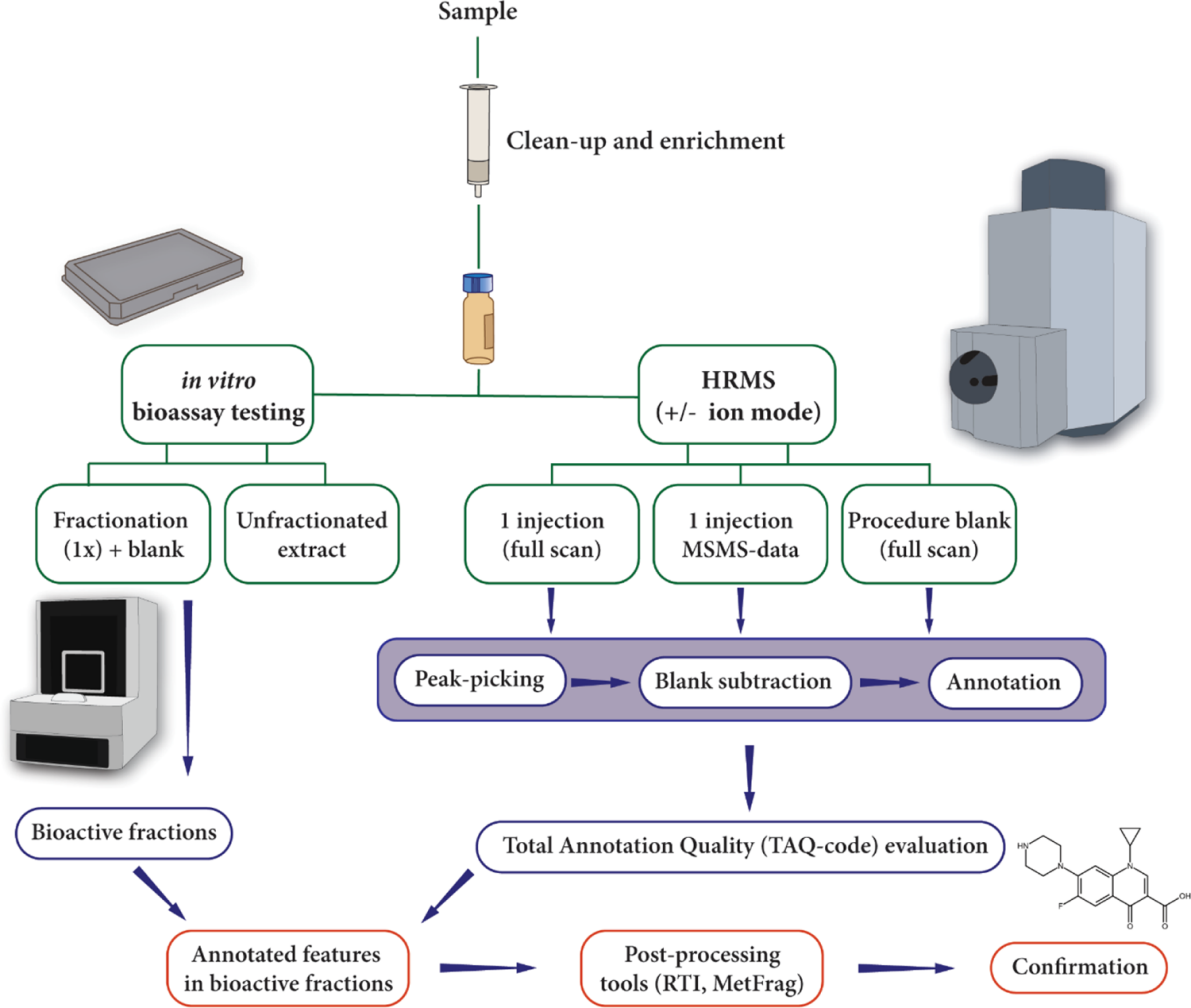
Robust workflow for EDA studies to identify bioactive contaminants. The integration of in vitro bioassays, HRMS, and state-of-the-art annotation approaches—such as comprehensive suspect screening, annotation quality control steps, and post-processing in silico tools (e.g., the RTI platform and MetFrag)—enhances the high-throughput identification of contaminants with toxicological effects.

In future work, improvements to the identification of multihalogenated compounds will be addressed. An optimal workflow for future EDA studies focusing on either environmental or human samples is provided in Figure 4, which will support the identification of the next generation of CECs using EDA, suspect, and nontarget screening. This workflow incorporates the experimental setup, data processing steps, and annotation of features including an automated annotation quality scoring system.

## Supporting Information Available

Additional information on the materials and methods of bioassay testing, concentrations of spiked compounds in FCS, MS(MS) acquisition and MetaboScape settings, classification details on TAQ-codes and their relation to Schymanski levels, number of features detected per matrix type, RTI platform box 1 CECScreen candidates processed with MetFrag, dose–response curve for T4 in the TTR-binding assay, dose–response curves for fipronil sulfone and bisphenol S in the TTR-binding assay, retention time comparison and MS/MS head-to-tail plots for fipronil sulfone and bisphenol S, elucidation of (4,5-dibromo-2-hydroxy-3,6-dimethylphenyl) hydrogen carbonate in MetFrag, calibration curves RTI system, MetFrag processed candidates and their best matches, and retention time shifts with and without the use of a splitter; processed feature tables (Zenodo, DOI:10.5281/zenodo.5657052) (PDF)
