# Supplementary material for: High-Performance Data Processing Workflow Incorporating Effect-Directed Analysis for Feature Prioritization in Suspect and Nontarget Screening
Source: Environ Sci Technol. 2022 Jan 20;56(3):1639–51. doi: 10.1021/acs.est.1c04168 (PMC8812114; doi:10.1021/acs.est.1c04168)
Supplement: Supplementary file 1 — es1c04168_si_001.pdf [file es1c04168_si_001.pdf]

Supporting Information

A High-Performance Data Processing Workflow Incorporating Effect-Directed Analysis for Feature Prioritization in Suspect and Nontarget Screening

Tim J.H. Jonkers [a,‡], Jeroen Meijer [a,b,‡], Jelle J. Vlaanderen [b], Roel C.H. Vermeulen [b], Corine J. Houtman [c], Timo Hamers [a], and Marja H. Lamoree [a,]*

[a] Department of Environment & Health, Faculty of Science, Amsterdam Institute of Molecular and Life Sciences, Vrije Universiteit Amsterdam, De Boelelaan 1085, 1081 HV Amsterdam, The Netherlands

[b] Institute for Risk Assessment Sciences (IRAS), Utrecht University, Yalelaan 2, 3584 CM Utrecht, the Netherlands

[c] The Water Laboratory, J.W. Lucasweg 2, 2031 BE Haarlem, The Netherlands

‡these authors contributed equally to this work

Pages: 25

Figures: 9

Tables: 11

Table of contents

	Page
**1. Materials and methods bioassay testing**	S3-S4
**1.1.** Antibiotics assay	S3
**1.2.** TTR-binding assay	S4
**Table S1.** Concentrations of the spiked thyroid hormone system disrupting compounds in fetal calf serum.	S5
**Table S2.** Full-scan MS acquisition configuration.	S5
**Table S3.** MS/MS acquisition configurations.	S6
**Table S4.** MS/MS collision energy ramp configurations.	S6
**Table S5.** T-ReX configuration in MetaboScape	S7
**Table S6.** TAQ-codes allocated to Schymanski confidence levels	S8
**Table S7.** The number of features detected in each matrix type (three injections).	S8
**Table S8.** The number of features detected in each matrix type (two injections).	S9
**Table S9.** The number of features detected in each matrix type (one injection).	S9
**Table S10.** A selection of bioactive fractions for each matrix type describing the combined number of features measured in positive- and negative ion mode that were related to the fractions.	S10-S15
**Table S11.** Retention Time Indices (RTI) platform box 1 CECscreen candidates that were processed using MetFrag.	S16
**Figure S1.** Classification details of the TAQ-code.	S17
**Figure S2.** Dose-response curve for T4 in the TTR-binding assay.	S17
**Figure S3.** Dose-response curves for fipronil sulfone and bisphenol S in the TTR-binding assay.	S18
**Figure S4.** Retention time comparison between the extracted-ion chromatograms of m/z 450.9263 [M-H]- (fipronil sulfone) and m/z 249.0227 [M-H]- (bisphenol S) of the serum sample and the analytical standards.	S18

**Figure S5.** Head-to-tail plots of the measured MS/MS spectra of m/z 450.9263 [M-H]- in serum and fipronil sulfone (A) and m/z 249.0227 [M-H]- in serum and bisphenol S (B).	S19
**Figure S6.** Extracted-ion chromatograms (EIC) of a potential bioactive compound with mass 338.8690 aligned with the bioassay response of the TTR-binding assay in serum and the corresponding MS/MS spectra matched to (4,5-dibromo-2-hydroxy-3,6-dimethylphenyl) hydrogen carbonate in MetFrag.	S20
**Figure S7.** Calibration curves between the measured retention times of a set of reference compounds and those of the RTI system.	S21
**Figure S8.** The best MetFrag MS/MS matches of the Retention Time Indices (RTI) platform box 1 CECscreen candidates	S22-S23
**Figure S9.** Extracted-ion chromatograms (EIC) of one of the compounds spiked in serum (6-OH-BDE99) with and without the use of a splitter.	S24
**References**	S25

1. Materials and methods bioassay testing

1.1. Antibiotics assay

The antibiotics assay was performed as described in earlier work,[1] with minor modifications. In short, *E. coli* FhuAT was cultivated overnight in LB-medium supplemented with chloramphenicol (25 μg/mL) at 37 ˚C. The following morning this culture was diluted 200 times in fresh medium and re-cultivated until it reached logarithmic growth in approximately four hours. Subsequently, the culture was diluted to an OD_{600} of 0.02. A two-fold dilution series of the DMSO extract was prepared in clear polystyrene F-bottom 96-well plate (Greiner Bio-One) in LB-medium from columns 1 to 10. The extract was tested in triplicate. Columns 11 and 12 were used for the negative and positive control (1 μg/mL vancomycin). The bacterial culture was added to the plate in aliquots of 50 μL per well and incubated in a plate shaker (Titramax 1000, Heidolph) at 37 ˚C and 600 rpm for approximately 4 hours. The absorbance (OD_{600}) read-out was performed, followed by the addition of resazurin (0.1 mg/mL) and the fluorescent read-out. Both read-outs were performed on a VarioSkan Flash luminometer (Thermo).

For the fractionated plates, 50 µl LB medium (25 µg/mL chloramphenicol) was added to wells A3-H12 and incubated at 600 rpm for at least 30 minutes at room temperature to dissolve the fractionated compounds. In wells A1-F1 a bioassay calibration curve was prepared with a two-fold dilution series of vancomycin (2.5 to 0.078 µg/mL) in LB supplemented with chloramphenicol. Wells G1-2 and H1-2 were used for negative and sterility controls, respectively. Further, the assay was performed as described above.

1.2. TTR-binding assay

The TTR-binding assay was performed as described in Hamers, et al. [2] with some modifications in incubation time and temperature. Instead of incubation for 2 hours at 4 ˚C, the plates were incubated at room temperature for 15 minutes in the dark on a plate shaker (Titramax 1000, Heidolph) at 600 rpm. The TTR-bind assay makes use of a fluorescent conjugate of T4 and FITC and repression of the binding of the T4-FITC complex to TTR results in a perturbed fluorescent signal which can be measured. The synthesis of the T4-FITC conjugate is described in detail by Ren and Guo [3]. The TTR-binding assays were performed in black 96-wells polystyrene nonbinding plates (Greiner Bio-One). For the unfractionated extract experiments 100 µl of a TRIS-buffer solution was added to all wells of the 96 wells plates after which 2 µl in duplo was added of the extract dilution series (1—3—10—30—100—300—1000—3000), negative control (DMSO), and a positive control (a calibration series of T4 without FITC (2—6—20—60—200—600—2000 nM)). Subsequently, 50 uL of the T4-FITC solution and 50 µL of the TTR solution was added to reach final volume of 200 µL with a concentration of 30nM and 110 nM of TTR and T4-FITC, respectively. Fluorescence (expressed as arbitrary fluorescence units (AFUs)) was measured after each time a reagent was added in the 96 wells plate at λ¬ex = 487 and λem =

528 using a Citation 5 image reader (BioTek) operated with the Gen 5 microplate reader and imager software (version 3.10; BioTek).

For the fractionated plates, 100 µl of TRIS-buffer solution was added to the plates and incubated at 600 rpm for at least 30 minutes to reconstitute the fractions. Subsequently, the T4 calibration dilutions were added in wells A1 – H2 as described above. Further the assay was performed as described previously.

Table S1. Concentrations of the spiked thyroid hormone system disrupting compounds in fetal calf serum.

Name	CAS no.	Concentration (µM)	Relative potencies*
TBBPA	79-94-7	0.043	1.5
2,4,6-TBP	118-79-6	0.033	2.4
5-OH-BDE47	602326-30-7	0.020	1.5
6-OH-BDE47	79755-43-4	0.115	0.68
6-OH-BDE99	297742-10-0	0.090	2.5
4-OH-CB107	152969-11-4	0.067	0.63
4-OH-CB187	158076-68-7	0.015	2.6

*The binding potencies relative to T4 were determined by Hamers, et al.[2]

Table S2. Full-scan MS acquisition configuration.

Parameter	Value
Source	Electrospray ionization (ESI)

Focus	Active
End Plate Offset (V)	500
Capillary (V)	4500
Nebulizer (Bar)	1.8
Dry Gas (l/min)	9
Dry Temp (˚C)	220
Mass range *from* (m/z)	50
Mass range to (m/z)	1300
Spectra Rate (Hz)	2

Table S3. MS/MS acquisition configurations.

Parameter	Value
**MS**	
Source	Electrospray ionization (ESI)
Focus	Active
End Plate Offset (V)	500
Capillary (V)	4500
Nebulizer (Bar)	1.8
Dry Gas (l/min)	9
Dry Temp (°C)	220
Mass range *from* (m/z)	50
Mass range to (m/z)	1300
Spectra Rate (Hz)	5
**MS/MS**	
Mode	Auto MS/MS (DDA)
Cycle Time (sec)	0.4
Absolute Threshold *per 1000 sum.* (cts)	600
Active Exclusion *exclude after* (spectra)	3
Active Exclusion *release after* (min)	0.1
Reconsider precursor (current I./previous I.)	5
Smart Exclusion	2x
Fixed MS/MS Acquisition (Hz)	5

Table S4. MS/MS collision energy ramp configurations.

Mass (m/z)	Width	Collision (eV)	Charge state
50	1	10	1
200	5	20	1
500	8	20	1
900	10	30	1
1300	12	30	1

Table S5. T-ReX configuration in MetaboScape for positive ion- and negative ion mode of the full bucket table.

Parameter	Positive ion mode	Negative ion mode
**Peak Detection**		
Intensity threshold (counts)	6000	6000
Minimum Peak Length (spectra)	11	11
Feature Signal	Intensity	Intensity
**Calibration**		
Mass recalibration *RT*	auto-detect	auto-detect
Mass recalibration *List*	Na Formate pos	Na Formate neg
**Recursive Feature Extraction**		
Minimum Peak Length *recursive* (spectra)*	7	7
Minimum # Features for Extraction (analysis)	1	1
**Bucket Filter**		
Presence of features in minimum # of analysis (analysis)	1	1
**MS/MS Import Configuration**		
Perform MS/MS import	Yes	Yes
MS/MS import method	Maxsum**	Maxsum**
Group by collision energy	Yes	Yes
**Ranges**		
Retention Time Range *Start* (min)	0.42	0.42
Retention Time Range *End* (min)	18	18
Mass Range *Start* (m/z)	50	50
Mass Range *End* (m/z)	1300	1300
Keep isotope pattern information from all analyses	Yes	Yes
**Ion Deconvolution**		
EIC Correlation	0.8	0.8
Primary Ion	[M+H]+	[M-H]-
Seed Ions	[M+Na]+; [M+K]+	[M+Cl]-
Common Ions	[M-H2O+H]+	[M-H2O-H]-

*=2/3rd of minimum peak length

** In case multiple MS/MS spectra exist for the same precursor, then the intensities of each MS/MS spectrum are summed and the one with the highest sum is selected.

Table S6. Annotation TAQ-codes categorized by the different identification confidence levels as proposed by Schymanski, et al. [4]. A threshold of 100 mSigma was applied as measure for the isotopic pattern accuracy, corresponding to a difference of ~10% between the measured and calculated isotopic pattern fit.[5] The additional levels (1-3) were applied for possible further prioritization on isotopic pattern accuracy if necessary. Features where parameter Z of the TAQ-code equals 0 (see Figure S1) were excluded from further data processing and as such were not assigned a confidence level.

Level 1	Level 2a	Level 2b	Level 3	Level 4*	Level 4	Level 5
2232Yes1	2132Yes1	2231Yes1	2031Yes1	2030Yes1	2030No1	-
2222Yes1	2122Yes1	2221Yes1	2021Yes1	2020Yes1	2020No1	
2212Yes1	2112Yes1	2211Yes1	2011Yes1	2010Yes1	2010No1	
	2032Yes1	2230Yes1	2131Yes1			
	2022Yes1	2220Yes1	2121Yes1			
	2012Yes1	2210Yes1	2111Yes1			
		2230No1	2130Yes1			
		2220No1	2120Yes1			
		2210No1	2110Yes1			
			2130No1			
			2120No1			
			2110No1			

*Level 4-features for which MS/MS spectra were recorded.

Table S7. The number of features detected in each matrix type (three injections), reported according to the identification confidence levels as proposed by Schymanski et al.[4]

Schymanski level	Effluent		Dust		Serum	
	Positive ion mode	Negative ion mode	Positive ion mode	Negative ion mode	Positive ion mode	Negative ion mode
1	16	3	6	3	0	7[a]
2a	25	2	26	26	33	33
2b	19	7	1	3	2	4
3	15	4	28	11	21	10
4*	134 (1036)	190 (642)	394 (5645)	156 (747)	307 (3326)	391 (3609)
4	601 (7993)	717 (2777)	2068 (39029)	950 (4453)	1344 (15686)	1649 (15019)
5	2490	3475	3932	8974	2737	2652

[a]Manually annotated; *Level 4-features for which MS/MS spectra were recorded. The total number of possible isomers identified through CECscreen is shown between parenthesis.

Table S8. The number of features detected in each matrix type (two injections), reported according to the identification confidence levels as proposed by Schymanski et al.[4]

Schymanski level	Effluent		Dust		Serum	
	Positive ion mode	Negative ion mode	Positive ion mode	Negative ion mode	Positive ion mode	Negative ion mode
1	16	2	6	3	0	7[a]
2a	25	2	26	26	34	33
2b	18	7	1	3	2	4
3	14	5	28	9	21	11
4*	140 (1162)	187 (616)	402 (5699)	107 (439)	306 (3341)	399 (3646)
4	602 (8056)	672 (2130)	2041 (37531)	658 (2744)	1318 (15334)	1602 (14645)
5	2419	3450	3884	9000	2692	2560

[a]Manually annotated; *Level 4-features for which MS/MS spectra were recorded. The total number of possible isomers identified through CECscreen is shown between parenthesis.

Table S9. The number of features detected in each matrix type (single injection), reported according to the identification confidence levels as proposed by Schymanski et al.[4]

Schymanski level	Effluent		Dust		Serum	
	Positive ion mode	Negative ion mode	Positive ion mode	Negative ion mode	Positive ion mode	Negative ion mode
1	15	2	5	3	0	7[a]
2a	25	3	26	25	33	33
2b	18	6	1	2	1	4
3	14	7	27	10	21	11
4*	135 (1103)	212 (740)	404 (5875)	95 (546)	307 (3299)	395 (3617)
4	501 (6265)	545 (2320)	1881 (34949)	425 (1936)	1235 (14229)	1452 (13424)
5	2299	3416	3752	8716	2703	2437

[a]Manually annotated; **Level 4-features for which MS/MS spectra were recorded. The total number of possible isomers identified through CECscreen is shown between parenthesis.

Table S10. A selection of bioactive fractions for each matrix type describing the combined number of features measured in positive- and negative ion mode that were related to the fractions. Features are ordered according to identification confidence level and the number of injections. The level 1 and 2 annotations are shown by name. The total number of possible isomers identified through CECscreen is shown between parenthesis.

Matrix	Fraction	No. injections	n	Level 1 (n)	Level 2a (n)	Level 2b (n)	Level 3 (n)	Level 4* (n)	Level 4 (n)	Level 5 (n)
Effluent	10	1	569	1H-Benzotriazole acid<br>2,5-Dimethylbenzenesulfonic acid<br>Amantadine<br>Lidocaine	2-Benzothiazolesulfonic acid<br>2,5-Dimethylbenzenesulfonic acid<br>3,4-Methylenedioxy-N-methylamphetamine (MDMA)<br>Leucylleucine	Ciprofloxacin-d8	-	20 (145)	52 (400)	488
		2	548	1H-Benzotriazole acid<br>2,5-Dimethylbenzenesulfonic acid<br>Amantadine	2-Benzothiazolesulfonic acid<br>3,4-Methylenedioxy-N-methylamphetamine (MDMA)	Ciprofloxacin-d8	-	22 (171)	52 (384)	466

			Lidocaine	Leucylleucine					
	3	541	1H-Benzotriazole acid<br>2,5-Dimethylbenzenesulfonic acid<br>Amantadine<br>Lidocaine	2-Benzothiazolesulfonic acid<br>3,4-Methylenedioxy-N-methylamphetamine (MDMA)<br>Leucylleucine	Ciprofloxacin-d8	-	20 (168)	62 (539)	451
11	1	521	Lidocaine	2,5-Dimethylbenzenesulfonic acid	1,3-diphenylguanidine<br>Ciprofloxacin-d8	1	16 (85)	47 (309)	453
	2	504	Lidocaine	-	1,3-diphenylguanidine<br>Ciprofloxacin-d8	1	14 (92)	59 (431)	427
	3	510	Lidocaine	-	1,3-diphenylguanidine<br>Ciprofloxacin-d8	1	15 (92)	59 (443)	432
16	1	434	-	Triethyl phosphate	Azithromycin<br>Azithromycin-13Cd3	2	20 (22)	58 (287)	351

	2	463	-	Triethyl phosphate	Azithromycin<br>Azithromycin-13Cd3	2	16<br>(20)	75<br>(327)	367
	3	469	-	Triethyl phosphate	Azithromycin<br>Azithromycin-13Cd3<br>Sulfamethoxazole	2	15<br>(20)	77<br>(337)	371
17	1	439	Bisoprolol A<br>Clozapine	S,R-Noscapine<br>Triethyl phosphate	Azithromycin<br>Azithromycin-13Cd3	21	56<br>(111)	354<br>(471)	354
	2	459	Bisoprolol A<br>Clozapine	S,R-Noscapine<br>Triethyl phosphate	Azithromycin<br>Azithromycin-13Cd3	18	71<br>(111)	362<br>(501)	362
	3	457	Bisoprolol A<br>Clozapine	S,R-Noscapine<br>Triethyl phosphate	Azithromycin<br>Azithromycin-13Cd3	16	73<br>(111)	360<br>(394)	360
28	1	192	DEET (N.N-Diethyl-3-methylbenzamide)<br>Losartan	Amitriptyline<br>Candesartan<br>Cetirizine<br>Climbazole<br>Methadone	Clarithromycin-N-methyl-13Cd3<br>Clarithromycin	2	12<br>(66)	53<br>(493)	115

					N-Desmethyl Clarithromycin					
		2	218	DEET (N.N-Diethyl-3-methylbenzamide) Losartan	Amitriptyline Candesartan Cetirizine Climbazole Methadone N-Desmethyl Clarithromycin	Clarithromycin-N-methyl-13Cd3 Clarithromycin	2	9 (49)	64 (458)	133
		3	229	DEET (N.N-Diethyl-3-methylbenzamide) Losartan	Amitriptyline Candesartan Cetirizine Climbazole Methadone N-Desmethyl Clarithromycin	Clarithromycin-N-methyl-13Cd3 Clarithromycin	2	9 (49)	66 (480)	142
Dust	39	1	540	-	N-Octyl-2-pyrrolidone	-	1	22 (310)	86 (1515)	430
		2	558	-	N-Octyl-2-pyrrolidone	-	1	17 (291)	102 (1405)	437

	3	573	-	N-Octyl-2-pyrrolidone	-	1	17 (291)	112 (1573)	442
40	1	370	-	Lauryl diethanolamide	-	1	16 (237)	85 (1803)	267
	2	379	-	Lauryl diethanolamide	-	1	15 (240)	88 (1654)	274
	3	386	-	Lauryl diethanolamide	-	1	15 (233)	95 (1819)	274
47	1	383	Diazinon	Dibutyl phthalate N-Dodecanoyl-N-methylglycine	-	-	13 (210)	70 (1295)	297
	2	399	Diazinon	Dibutyl phthalate N-Dodecanoyl-N-methylglycine	-	-	14 (212)	80 (1290)	302
	3	409	Diazinon	Dibutyl phthalate N-Dodecanoyl-N-methylglycine	-	-	13 (204)	88 (1288)	305

63	1	333	-	Anthranilic acid	-	1	13 (223)	57 (632)	261
	2	366	-	-	-	1	13 (187)	65 (692)	287
	3	370	-	-	-	1	12 (186)	69 (666)	288
64	1	381	-	Anthranilic acid Oleyl sarcosine	-	-	11 (172)	70 (1116)	298
	2	407	-	Anthranilic acid	-	-	14 (178)	77 (1276)	315
	3	409	-	Anthranilic acid	-	-	14 (164)	84 (1303)	310
72	1	313	-	-	-	1	12 (122)	52 (1018)	248
	2	347	-	-	-	1	15 (152)	67 (1366)	264

		3	347	-	-	-	1	11 (117)	71 (1349)	264
Serum	17	1	179	-	4-Hydrobenzoic acid<br>Azelaic acid<br>Indolacetic acid	Bisphenol S	1	23 (292)	58 (673)	93
		2	200	-	4-Hydrobenzoic acid<br>Azelaic acid<br>Indolacetic acid	Bisphenol S	1	24 (299)	67 (765)	104
		3	196	-	4-Hydrobenzoic acid<br>Azelaic acid<br>Indolacetic acid	Bisphenol S	1	22 (295)	66 (714)	103
	21	1	208	-	Decanedioic acid<br>Hippuric acid	-	1	34 (293)	93 (1441)	78
		2	228	-	Decanedioic acid<br>Hippuric acid	-	1	36 (315)	104 (1785)	85
		3	237	-	Decanedioic acid<br>Hippuric acid	-	1	36 (313)	110 (1685)	88

50	1	22<br>3	-	Fipronil sulfone<br>LPC 16:0<br>LPC 18:1	-	-	12<br>(70)	60<br>(485)	148
	2	23<br>6	-	Fipronil sulfone<br>LPC 16:0<br>LPC 18:1	-	-	16<br>(98)	59<br>(367)	158
	3	23<br>3	-	Fipronil sulfone<br>LPC 16:0<br>LPC 18:1	-	-	17<br>(104)	62<br>(388)	151
51	1	35<br>7	-	LPC 16:0<br>LPC 18:1	-	3	13<br>(105)	58<br>(331)	281
	2	35<br>8	-	LPC 16:0<br>LPC 18:1	-	3	15<br>(116)	64<br>(375)	274
	3	36<br>2	-	LPC 16:0<br>LPC 18:1	-	3	15<br>(120)	65<br>(381)	277
61	1	20<br>6	-	-	-	3	10<br>(34)	46<br>(313)	147
	2	21<br>2	-	-	-	3	10<br>(34)	48<br>(300)	151
	3	22<br>0	-	-	-	3	10<br>(34)	57<br>(347)	150

62	1	20<br>7	-	-	-	2	12<br>(39)	46<br>(327)	147
	2	21<br>2	-	-	-	3	11<br>(33)	48<br>(300)	150
	3	22<br>2	-	-	-	3	12<br>(39)	56<br>(358)	151

Table S11. Retention Time Indices (RTI) platform box 1 CECscreen candidates of feature number 1522, 3837, and 4255 for effluent dust and serum, respectively, which were processed using MetFrag.

Matrix (feature #)	Mass (m/z)	Name	CAS#	RT (min)	Pred. RT (min)	Δ RT (min)
Effluent (1522)	250.1808	N-Desmethyltramadol	1018989-94-0	2.03	2.63	0.6
	250.1808	O-Desmethyltramadol	73986-53-5	2.03	2.48	0.45
	250.1808	Procinolol	27325-36-6	2.03	2.91	0.88
Dust (3837)	186.1854	2-isopropyl-N,N,2,3-tetramethylbutyramide	51115-69-6	8.86	8.79	0.07
	186.1854	N-Methylneodecanamide	105726-67-8	8.86	9.21	0.35
	186.1854	N,N,3,5,5-pentamethylhexanamide	80480-32-6	8.86	7.67	1.19
	186.1854	N-ethyl-2-isopropyl-2,3-dimethylbutyramide	51115-71-0	8.86	9.1	0.24
	186.1854	N,2,2-triethyl-3-methylbutyramide	51115-73-2	8.86	8.92	0.06
	186.1854	Valdipromide	52061-73-1	8.86	9.29	0.43
Serum (4255)	313.2373	3-(2-hydroxyoctyl)oxiran-2-octanoic acid	47244-76-8	11.89	11.92	0.03
	313.2373	1,18-octadecanedioic acid	871-70-5	11.89	11.67	0.22
	313.2373	Isononyl hydrogen azelate	71850-13-0	11.89	13.08	1.19
	313.2373	Tetradecylsuccinic acid	2530-30-5	11.89	11.84	0.05

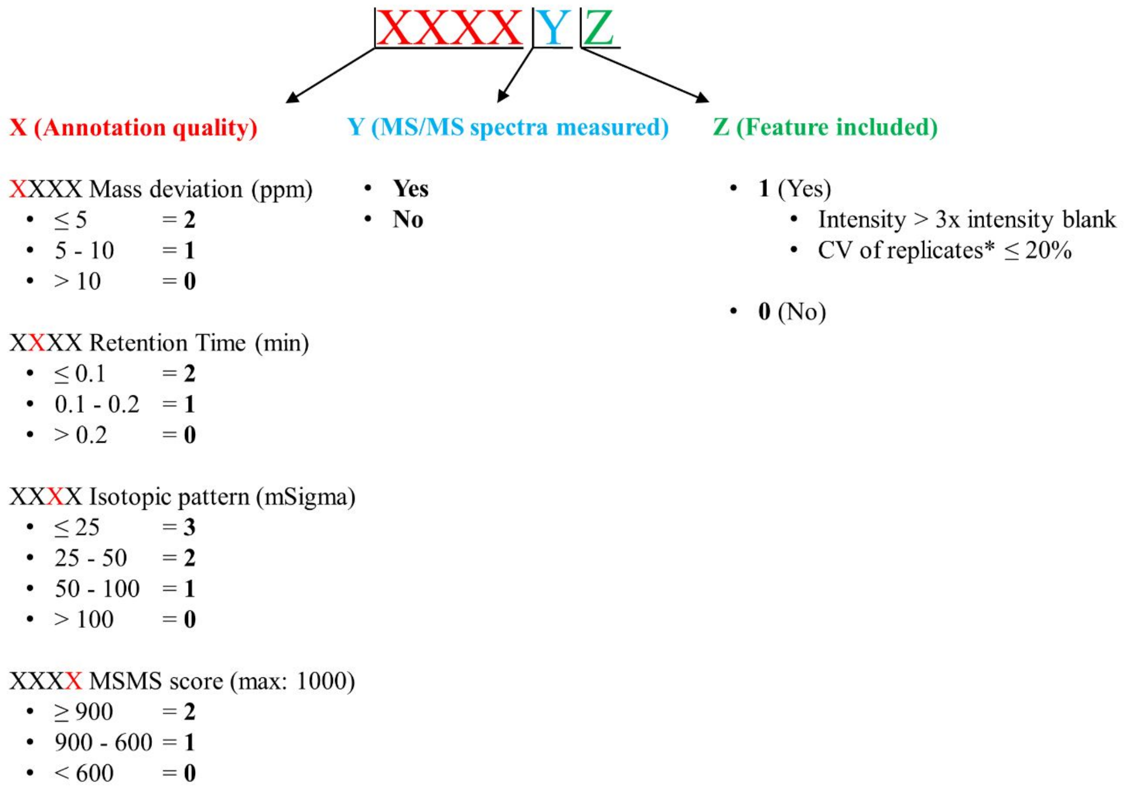

* Only when multiple technical replicates are used

Figure S1. Classification details of the TAQ-code including the annotation quality (RED), whether an MS/MS spectra was measured for that feature (BLUE) and whether the feature was included based on the criteria (GREEN). The retention time score is based on the measured retention times of standards.

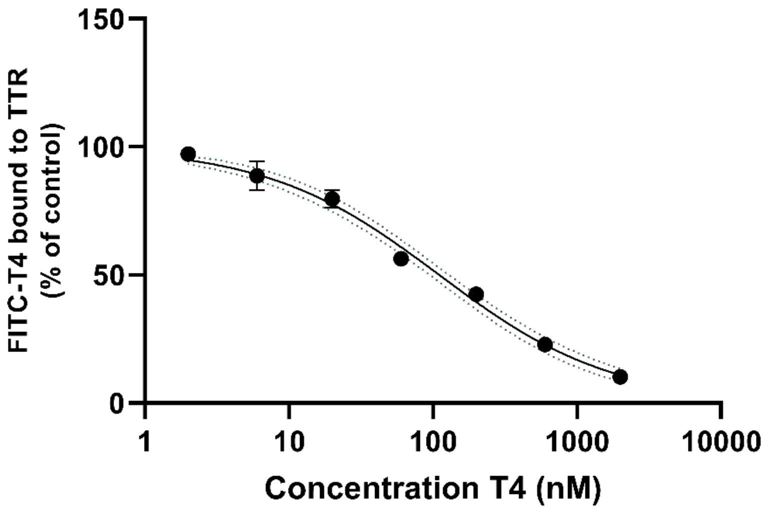

Figure S2. Dose-response curve for T4 in the TTR-binding assay. The linear response of the sigmoidal curve starts around 80% of FITC-T4 bound to TTR compared to the control.

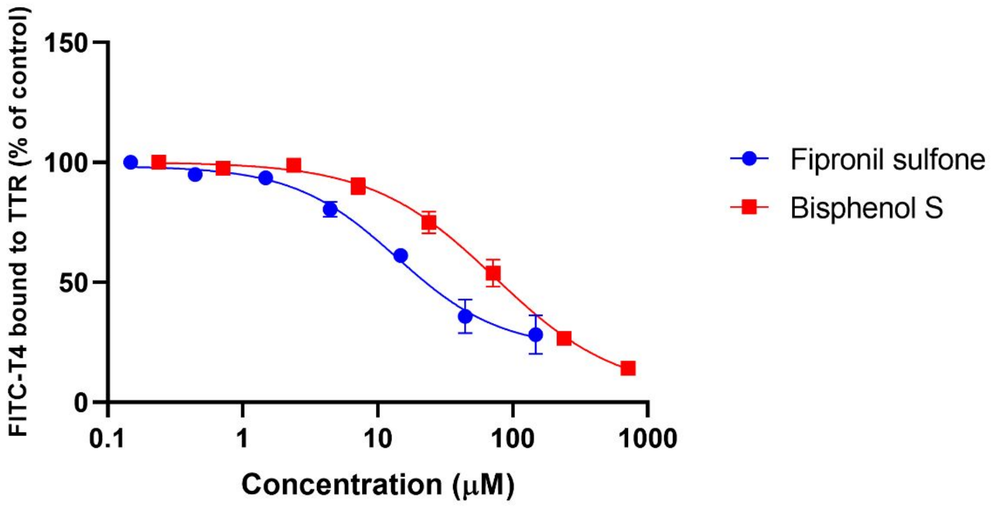

Figure S3. Dose-response curves for fipronil sulfone and bisphenol s in the TTR-binding assay. The IC50-value of fipronil sulfone and bisphenol s were determined to be 14 and 73 µM, respectively.

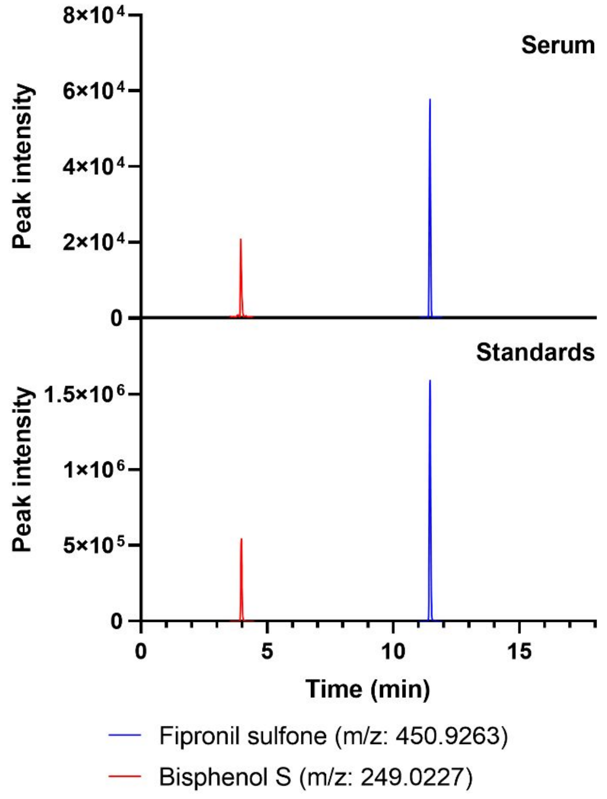

Figure S4. Retention time comparison between the extracted-ion chromatograms of m/z 450.9263 $[M-H]^-$ (fipronil sulfone) and m/z 249.0227 $[M-H]^-$ (bisphenol S) of the serum sample and the analytical standards. The standards were injected at a concentration of 125 ppb.

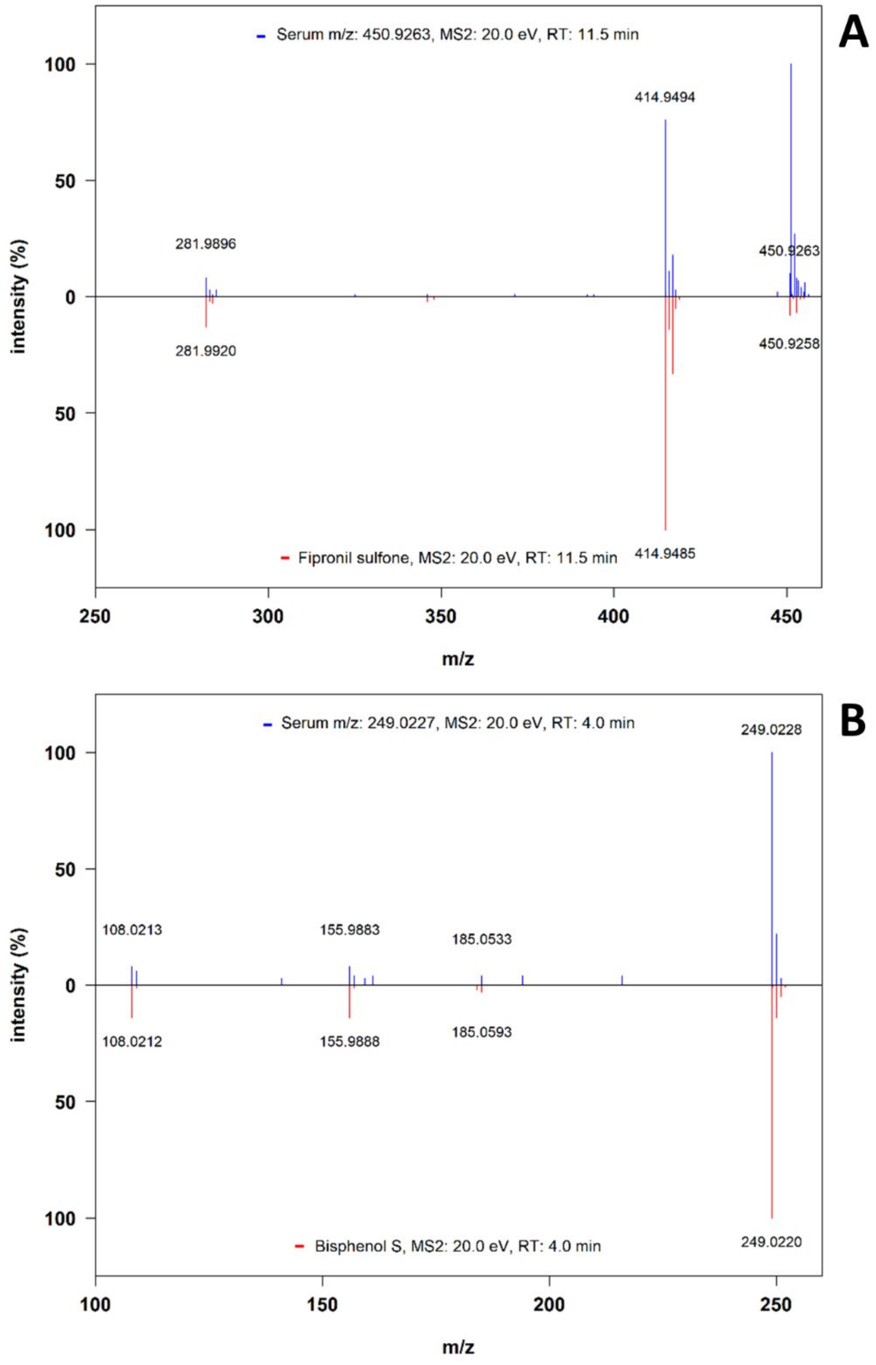

Figure S5. Head-to-tail plots of the measured MS/MS spectra of m/z 450.9263 [M-H]- in serum and fipronil sulfone (A) and m/z 249.0227 [M-H]- in serum and bisphenol S (B). All MS/MS spectra were measured using collision-induced dissociation (CID) at 20.0 eV.

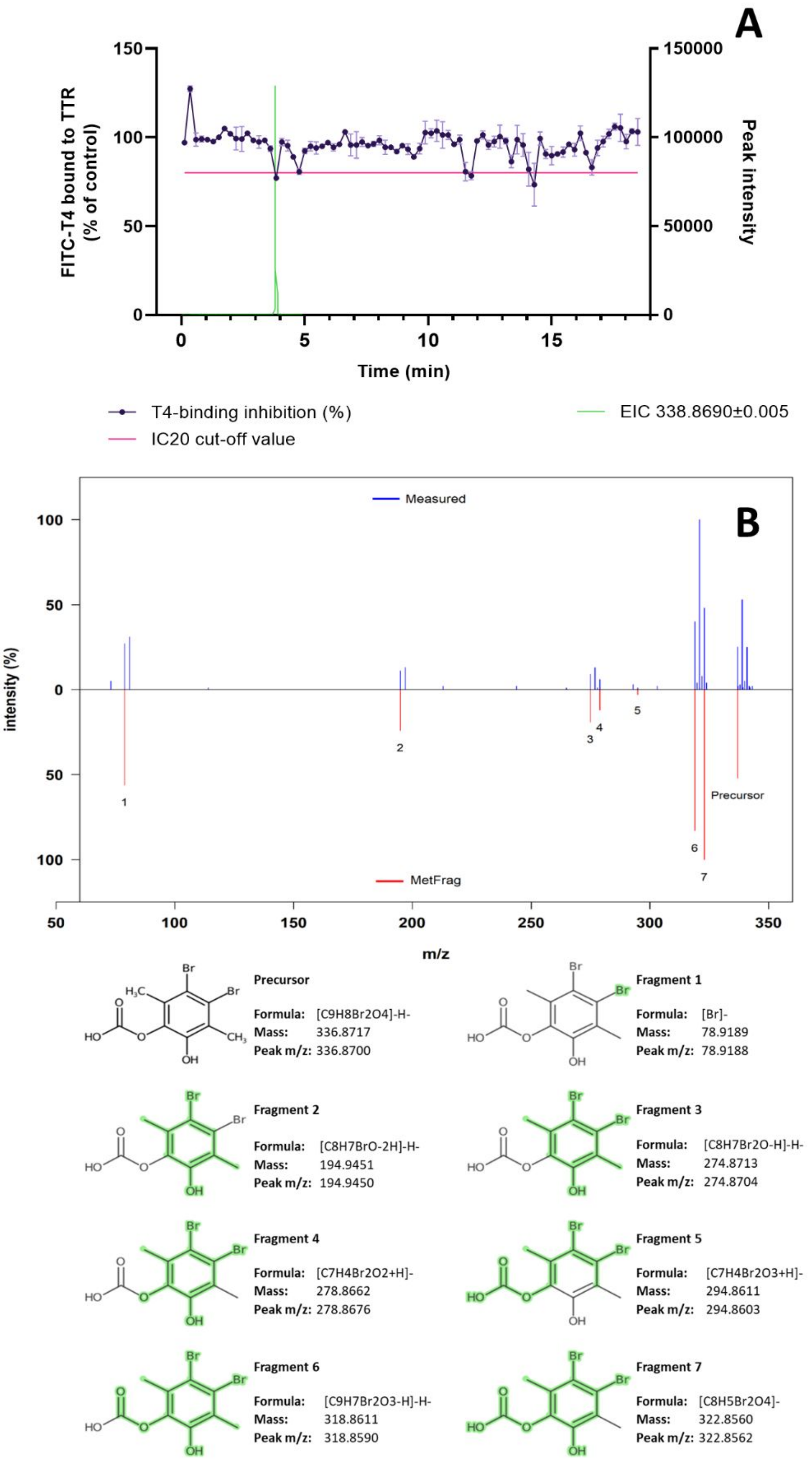

Figure S6. Extracted-ion chromatogram (EIC) of m/z 338.8690 $[M-H]^-$ aligned with the TTR-binding assay response of serum (A), and the corresponding MS/MS spectrum matched to (4,5-dibromo-2-hydroxy-3,6-dimethylphenyl) hydrogen carbonate in MetFrag (B).

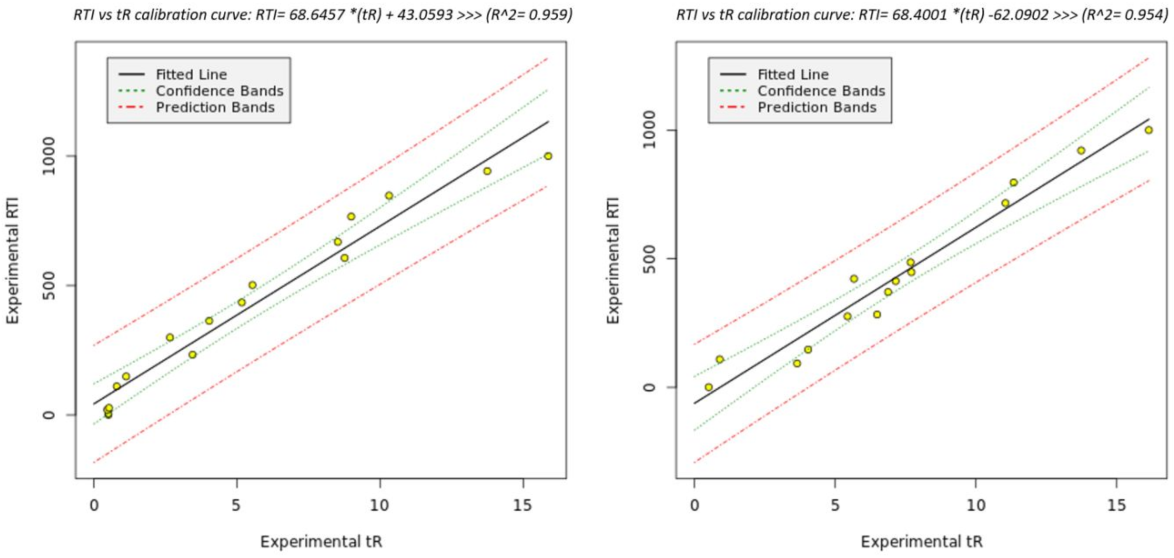

Figure S7. Calibration curves between the measured retention times of a set of reference compounds and those of the RTI system.

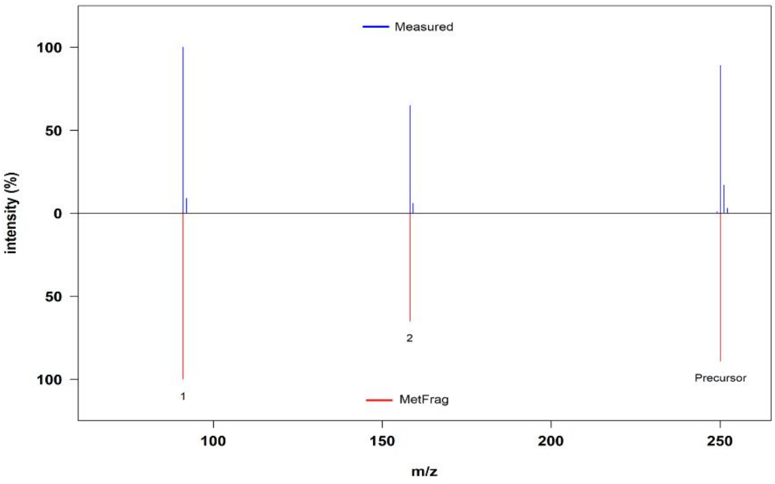

A

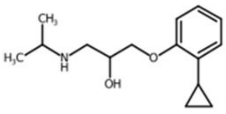

Precursor

Formula: [C15H23N1O2]+H+
Mass: 250.1802
Peak m/z: 250.1803

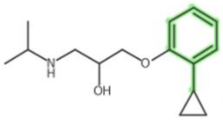

Fragment 1

Formula: [C7H5+H]+H+
Mass: 91.0543
Peak m/z: 91.0544

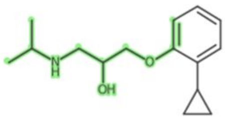

Fragment 2

Formula: [C8H15NO2]+H+
Mass: 158.1176
Peak m/z: 158.1177

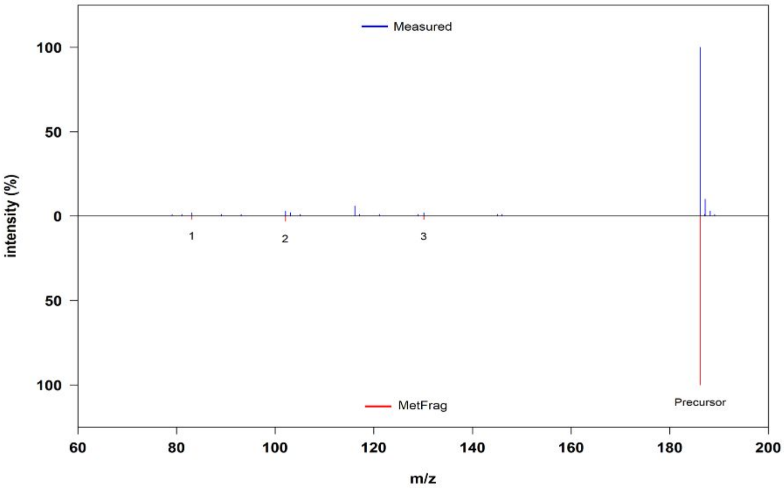

B

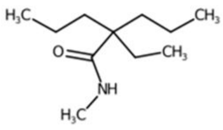

Precursor

Formula: [C15H23N1O2]+H+
Mass: 186.1852
Peak m/z: 186.1854

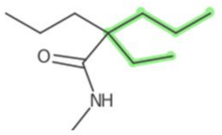

Fragment 1

Formula: [C6H12-H]+
Mass: 83.0856
Peak m/z: 83.0858

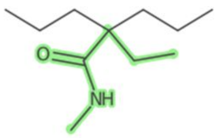

Fragment 2

Formula: [C5H9NO+2H]+H+
Mass: 102.0914
Peak m/z: 102.0910

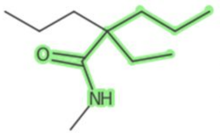

Fragment 3

Formula: [C7H13NO+2H]+H+
Mass: 130.1227
Peak m/z: 130.1213

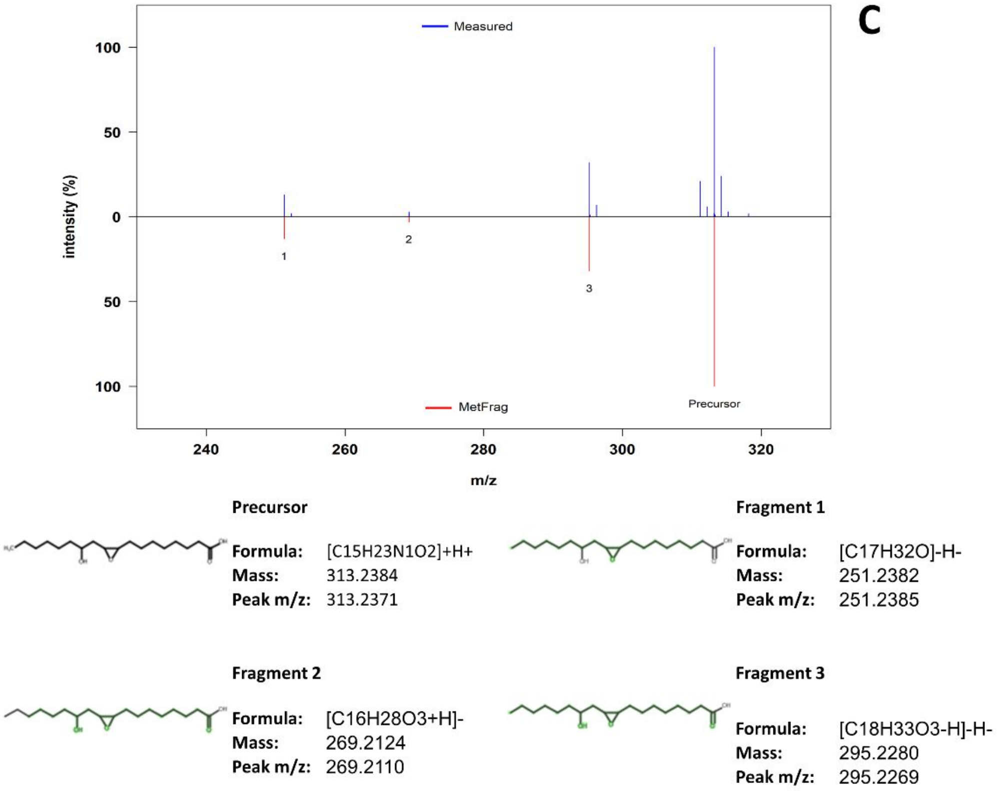

Figure S8. The best MetFrag MS/MS matches of the Retention Time Indices (RTI) platform box 1 CECscreen candidates of feature numbers 1522, 3837 and 4255 for effluent (A), dust (B) and serum (C), respectively. NB the fragments are presented as direct output from MetFrag. The tool may predict unrealistic fragments and should be manually interpreted. Dust feature number 3837 (B) included six candidates from Box 1 with similar predicted retention times except for N,N,3,5,5-pentamethylhexanamide (ΔRT =1.19 minutes). In MetFrag, two of these compounds (N,N,3,5,5-pentamethylhexanamide and N-Methylneodecanamide) had the highest prediction scores with three matching fragments. Taking the retention time prediction into consideration, the most likely annotation was N-Methylneodecanamide. Box 1 candidates of serum feature number 4255 (C) included 3-(2-hydroxyoctyl)oxiran-2-octanoic acid (compound 1), 1,18-octadecanedioic acid (compound 2), isononyl hydrogen azelate (compound 3), and tetradecylsuccinic acid (compound 4). There was a small difference in predicted retention times of these isomers, except for compound 3 (ΔRT =1.19 minutes between measured and predicted). MetFrag gave comparable prediction scores for each candidate because the structures were similar. However, for compound 1 and compound 4 three fragments and for compound 2 and

compound 3 two fragments were matched, respectively. If the difference in the experimental and predicted retention time is considered, compound 1 has the highest probability of being the correct annotation.

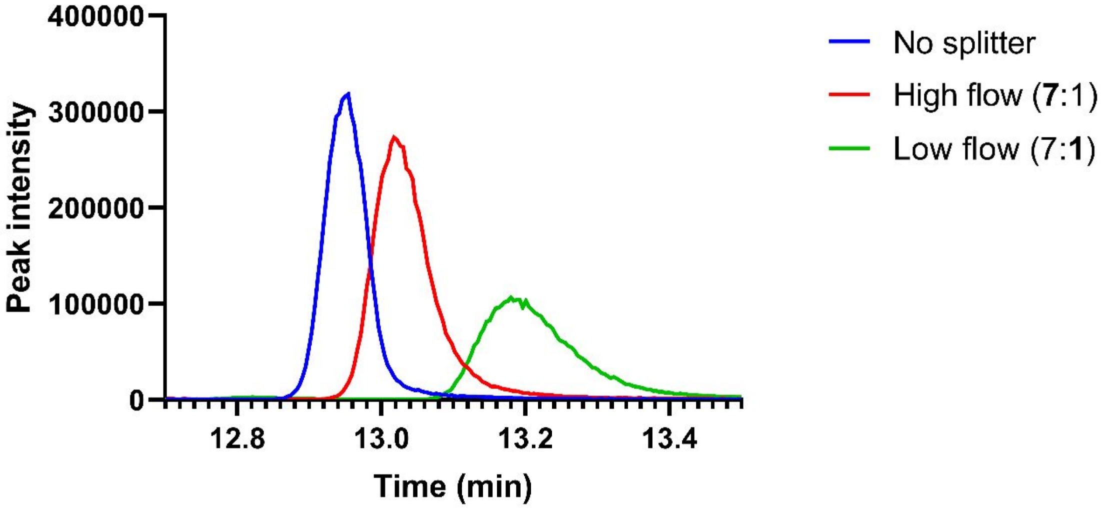

Figure S9. Extracted-ion chromatograms (EIC) of one of the compounds spiked in serum (6-OH-BDE99) with and without the use of a splitter. The split ratio that was used in this experiment was 7:1, which resulted in a high flow of ± 438 µl/min and a low flow of 62 µl/min after the splitter. 6-OH-BDE99 was measured using both the low flow- and the high flow port. As a result of the splitter there is a slight shift in retention time to the right and in peak width and height. These modifications should be taken into consideration when using the splitter in EDA studies because it might introduce larger errors in the alignments of the chromatogram and the fractionated extract bioassay results.

References

1. Jonkers, T. J.; Steenhuis, M.; Schalkwijk, L.; Luirink, J.; Bald, D.; Houtman, C. J.; Kool, J.; Lamoree, M. H.; Hamers, T., Development of a high-throughput bioassay for screening of antibiotics in aquatic environmental samples. *Science of The Total Environment* **2020**, 139028.
2. Hamers, T.; Kortenkamp, A.; Scholze, M.; Molenaar, D.; Cenijn, P. H.; Weiss, J. M., Transthyretin-binding activity of complex mixtures representing the composition of thyroid-hormone disrupting contaminants in house dust and human serum. *Environmental health perspectives* **2020,** *128*, (1), 017015.
3. Ren, X. M.; Guo, L.-H., Assessment of the binding of hydroxylated polybrominated diphenyl ethers to thyroid hormone transport proteins using a site-specific fluorescence probe. *Environmental science & technology* **2012,** *46*, (8), 4633-4640.
4. Schymanski, E. L.; Jeon, J.; Gulde, R.; Fenner, K.; Ruff, M.; Singer, H. P.; Hollender, J., Identifying small molecules via high resolution mass spectrometry: communicating confidence. In ACS Publications: 2014.
5. Pelander, A.; Decker, P.; Baessmann, C.; Ojanperä, I., Evaluation of a high resolving power time-of-flight mass spectrometer for drug analysis in terms of resolving power and acquisition rate. *Journal of the American Society for Mass Spectrometry* **2011,** *22*, (2), 379-385.
